# Metagenomics Reveals Pervasive Bacterial Populations and Reduced Community Diversity across the Alaska Tundra Ecosystem

**DOI:** 10.3389/fmicb.2016.00579

**Published:** 2016-04-25

**Authors:** Eric R. Johnston, Luis M. Rodriguez-R, Chengwei Luo, Mengting M. Yuan, Liyou Wu, Zhili He, Edward A. G. Schuur, Yiqi Luo, James M. Tiedje, Jizhong Zhou, Konstantinos T. Konstantinidis

**Affiliations:** ^1^School of Civil and Environmental Engineering, Georgia Institute of Technology, AtlantaGA, USA; ^2^Center for Bioinformatics and Computational Genomics, Georgia Institute of Technology, AtlantaGA, USA; ^3^School of Biology, Georgia Institute of Technology, AtlantaGA, USA; ^4^Department of Microbiology and Plant Biology, Institute for Environmental Genomics, University of Oklahoma, NormanOK, USA; ^5^Department of Biological Sciences, Northern Arizona University, FlagstaffAZ, USA; ^6^Center for Microbial Ecology, Michigan State University, East LansingMI, USA; ^7^Earth Science Division, Lawrence Berkeley National Laboratory, BerkeleyCA, USA; ^8^State Key Joint Laboratory of Environment Simulation and Pollution Control, School of Environment, Tsinghua UniversityBeijing, China

**Keywords:** tundra, soil microbiology, metagenomics, ecosystem ecology, environmental science, permafrost, climate change, microbial diversity

## Abstract

How soil microbial communities contrast with respect to taxonomic and functional composition within and between ecosystems remains an unresolved question that is central to predicting how global anthropogenic change will affect soil functioning and services. In particular, it remains unclear how small-scale observations of soil communities based on the typical volume sampled (1–2 g) are generalizable to ecosystem-scale responses and processes. This is especially relevant for remote, northern latitude soils, which are challenging to sample and are also thought to be more vulnerable to climate change compared to temperate soils. Here, we employed well-replicated shotgun metagenome and 16S rRNA gene amplicon sequencing to characterize community composition and metabolic potential in Alaskan tundra soils, combining our own datasets with those publically available from distant tundra and temperate grassland and agriculture habitats. We found that the abundance of many taxa and metabolic functions differed substantially between tundra soil metagenomes relative to those from temperate soils, and that a high degree of OTU-sharing exists between tundra locations. Tundra soils were an order of magnitude less complex than their temperate counterparts, allowing for near-complete coverage of microbial community richness (~92% breadth) by sequencing, and the recovery of 27 high-quality, almost complete (>80% completeness) population bins. These population bins, collectively, made up to ~10% of the metagenomic datasets, and represented diverse taxonomic groups and metabolic lifestyles tuned toward sulfur cycling, hydrogen metabolism, methanotrophy, and organic matter oxidation. Several population bins, including members of *Acidobacteria*, *Actinobacteria*, and *Proteobacteria*, were also present in geographically distant (~100–530 km apart) tundra habitats (full genome representation and up to 99.6% genome-derived average nucleotide identity). Collectively, our results revealed that Alaska tundra microbial communities are less diverse and more homogenous across spatial scales than previously anticipated, and provided DNA sequences of abundant populations and genes that would be relevant for future studies of the effects of environmental change on tundra ecosystems.

## Introduction

Terrestrial soil systems are residence to some of the most functionally and taxonomically diverse microbial communities known (Torsvik et al., 1990; Whitman et al., 1998; Curtis et al., 2002; Handelsman et al., 2007). An increasing amount of attention has been directed toward these communities due to human dependence on soil productivity for food and fiber, the ecosystem services they provide (e.g., water quality, nutrient cycling), and their role in producing and consuming greenhouse gasses. Soil systems are estimated to contain more carbon than aboveground plant biomass and atmospheric pools combined in the form of degradable soil organic matter (or SOM) (Grosse et al., 2011). Higher land temperatures are expected to cause the release of considerable amounts of CO_2_ and CH_4_ to the atmosphere (Heimann and Reichstein, 2008; Mackelprang et al., 2011; McCalley et al., 2014), primarily through the microbially mediated degradation of SOM. Thus, there is an imminent need to further understand the role of soil microbes in the cycling of SOM C and other major elements, both to improve climate change predictions and possibly to mitigate climate change impacts through changes in land management practices. Tundra SOM is particularly sensitive to climate change (Jorgenson et al., 2010; Grosse et al., 2011) because low temperatures and saturated soil conditions protect organic C from microbial decomposition (McGuire et al., 2010; Lee et al., 2012; Pries et al., 2012). Furthermore, more than 50% of global soil organic C is stored in northern tundra permafrost, which only accounts for approximately 16% of the global soil area (Tarnocai et al., 2009). It is projected that permafrost may recede by 30–70% toward the end of the 21st century due to increasing temperatures (Schuur and Abbott, 2011; Lawrence et al., 2012), likely resulting in enormous terrestrial ecosystem C loss.

Our ability to predict soil ecosystem functioning and resilience and to manipulate terrestrial soils for enhanced C sequestration is hindered, at least partially, by the enormous diversity and as yet uncultivated status of soil microorganisms (Whitman et al., 1998; Handelsman et al., 2007). Several recent studies have employed ‘omics’ methodologies (i.e., metagenomics, metatranscriptomics, metaproteomics, etc.) to characterize microbes and their metabolisms present in tundra locations and have successfully assembled novel population bins (i.e., consensus genome assembly from a natural population), representing organisms relevant to CH_4_ and CO_2_ release, a feat that opens up new opportunities to directly study the *in situ* response of specific organisms (Mondav et al., 2014; Hultman et al., 2015). However, much remains unknown about what prokaryotic taxa dominate tundra, how much they vary in abundance across distant sites with similar environmental features, what pathways they encode and perhaps more importantly, what abiotic and biotic factors control the activity of these pathways and how environmental changes will affect that activity. It is also unclear how the genetic information present in the small volume of soil typically sampled (1–2 g) by these previous surveys relates to ecosystem-scale responses and processes. For this, surveys that analyze multiple replicated samples are needed.

Our team has been performing warming manipulations that raised *in-situ* temperatures by 2–5°C, simulating the effect of future climate change, for active layer soil atop permafrost at the Carbon in Permafrost Experimental Heating Research (CiPEHR) site (Alaska, USA; “AK site”) (Natali et al., 2011, 2014; Zhou et al., 2012). In total, 11 soils from the CiPEHR, AK site were collected from 15 to 25 cm depths in 2010, after about 1.5-year of experimental warming. Only minor differences were observed between warming and control plots at the DNA level (metagenomics) for these samples (Xue et al., 2016), presumably due to the slow growth kinetics of tundra microbes. Here, we took advantage of the well-replicated sequence datasets available, and pooled them together in order to robustly address the following objectives: (1) evaluate the biogeography of microorganisms in tundra soils at the 16S rRNA gene level as well as at the individual population (whole-genome) level. The latter is a better proxy for species since it circumvents the limitations of 16S rRNA gene related to high sequence conservation and represents an important and highly resolved unit of microbial communities (Caro-Quintero and Konstantinidis, 2012). (2) Identify the similarities in taxonomic and functional gene composition in active-layer soil sampled from various Alaskan tundra locations, using soil communities from temperate locations for comparison. And, (3) assess how these tundra microbial populations might respond to major environmental perturbations such as fire events. Our work identified several highly abundant (>1% of total community) populations that are ubiquitous across the tundra ecosystem in Alaska and thus, represent important members of the indigenous communities. It also revealed that these populations are highly dynamic, and can undergo rapid genomic alternations in gene content upon major environmental perturbations.

## Materials and Methods

### Site Description and Sampling

The CiPEHR site was established in September 2008 at a moist acidic tundra area in Interior Alaska near Denali National Park in the Eight Mile Lake region (63°52′59″N, 149°13′32″W). The experimental plots were located in the discontinuous permafrost region where permafrost thaw has been observed in the past several decades. Experimental design and site description were described in detail previously (Natali et al., 2011). Generally, three experimental blocks were located approximately 100 m away from each other. In each block, two snow fences were constructed about 5 m apart in the winter. The winter warming treatment plots were located 5 m back from the leeward side of the snow fences, while the paired control plots were at the windward side of snow fences. Soil temperature was increased in the winter warming plots due to thicker snow cover on soil surface and lower wind strength. The snow fences were removed in the spring before the snow melting to uniform hydraulic condition in both winter warming and control treatments. From 1976 to 2009, mean monthly temperature in the field ranged from -16°C in December to 15°C in July, with an annual mean temperature of -1.0°C. The average annual precipitation was 378 mm. Only C_3_ plant species were observed in this area. Dominant species include *Eriophorum vaginatum*, *Vaccinium uliginosum*, some other vascular species, non-vascular feather moss and lichen. In the experimental plots, the upper 0.45–0.65 m soil was rich in organic C materials and below was mineral soil with a mixture of glacial till and windblown loess. The active layer depth was about 50 cm.

Eleven soil cores, five from control plots and six from warmed plots (there were originally twelve soil samples – one control soil was discarded as it was deemed contaminated with Oklahoma soil DNA, and thus, incomparable to other soil samples), were taken using electric drills in destructive sampling plots at the six snow fences in the beginning of 2010 growing season (May), one and half year after the initial of winter warming treatment. Soil fractions of 15–25 cm from ground surface were used for this study.

The experimental warming site at Oklahoma is located at the Kessler Farm Field Laboratory (KFFL, OK site) at the Great Plain Apiaries in McClain County, Oklahoma, USA (34°58′54″N, 97°31′14″W). Block design was applied in this field experiment and adjacent blocks were 2 or 5 m apart. The six plots in each of the four blocks represent control, warming, half or double precipitation treatments and the combination manipulation of these treatments. Additionally, every southern half subplot was clipped twice a year to create a coupled clipping effect. Only warming and control treatments without water and clipping effects were included in this study. Beginning in early 2009, the soil temperature in the warming treatment plots was increased by the Kalglo MRM-1215 120 V, 1500 W, 65 inch-long electric infrared radiators (Kalglo Electronics, Bethlehem, PA, USA) fixed at 1.5 m above the ground surface at the center of each plot. In control plots, wood “dummy” heaters were used to simulate shading effect in warming plots. The herbivores were excluded at this site to prevent grazing. The plant community undergoes a relatively rapid secondary succession in this site and new species occur every year, causing gradually change in plant community structure. Although both C_3_ and C_4_ plants were observed, C_3_ species have dominated in recent years. Plant biomass peaks twice in late spring and early autumn every year. C_3_ grass *Bromus arvensis* and C_3_ forb *Vicia sativa* dominated the site in April 2010, while C_3_ forb *Ambrosia trifida*, *Solanum carolinense* and C_4_ grass *Tridens flavus* prevailed in August 2010. Based on Oklahoma Climatological Survey from 1948 to 1999, the mean annual temperature in this site was 16.3°C, with the lowest monthly mean of 3.3°C in January and the highest of 28.1°C in July. The precipitation was unevenly distributed annually, which peaked in May and June (240 mm) and reached a low in January and February (82 mm) with an annual mean of 967 mm (Zhou et al., 2012). Soils from the layer 0–15 cm in four warming plots and four control plots were sampled in the OK, USA site using a standard soil core (2.5 cm in diameter) in October 2010. All samples were transported to the laboratory and stored at -80°C immediately until analyses. Any observable plant root materials were picked out before the soil was processed. Fungal community composition of CiPEHR and KFFL soil communities has been addressed previously (Penton et al., 2013). Environmental indices for KFFL and CiPEHR, and the associated methods, are provided in Xue et al. (2016); a summary of select soil measurements (including C%, N%, SOM fractions, pH, and bulk density) is given in **Supplementary Table S1**.

### DNA Extraction of Soil Microbial Community

Soil DNA was extracted using a PowerMax Soil DNA Isolation Kit (MO BIO Laboratories, Inc., Carlsbad, CA, USA) according to manufacturer’s protocol. DNA quality was assessed based on spectrometry absorbance at wavelengths of 230, 260, and 280 nm detected by a NanoDrop ND-1000 Spectrophotometer (NanoDrop Technologies Inc., now NanoDrop Products by Thermo Fisher Scientific). The absorbance ratios of 260/280 nm were around 1.8, and of 260/230 nm were larger than 1.7. Finally, DNA was quantified by Pico Green using a FLUOstar OPTIMA fluorescence plate reader (BMG LabTech, Jena, Germany) and used for gene array labeling and sequencing library preparation.

### Illumina MiSeq Sequencing Protocol

The 16S rRNA library was prepared using methods introduced by Caporaso et al. (2011, 2012). In brief, extracted DNA samples were diluted to 2.5 ng/μL for PCR amplification. The primer sets used to amplify the V4 region of 16S rRNA genes were constructed to adapt the barcode Illumina MiSeq (Caporaso et al., 2011): the forward PCR primer contains an Illumina adapter sequence, followed by a forward primer pad, a forward primer link and then the 515 forward primer; besides above elements for the reverse primer (806 reverse primer was used), the reverse PCR primer also contains a sample-unique barcode sequence inserted between the reverse Illumina adapter and the reverse primer pad sequences for parallel sequencing of a sample set. The 25 μL PCR reaction system and condition was as documented in Caporaso et al. (2011). Only one PCR reaction was performed per sample. After the amplification the products were quantified using PicoGreen on a FLUOstar OPTIMA fluorescence plate reader (BMG LabTech). 100 ng PCR products from each sample were combined into one tube, ran on a 1% agarose gel at 100 V for 45 min, and purified through QIAquick Gel Extraction Kit (Qiagen) column. The purified sample was quantified again using PicoGreen by triplicates, ensured the accuracy of library concentration. Then the pooled sample was diluted to 2 nM. Ten microliter of 0.1 N NaOH was then added into 10 μL sample DNA for denaturation. Then the denatured DNA was diluted to 6 pM and mixed with equal volume of 6 pM Phix library in order to increase sequence diversity. Finally, the mixture (600 μL) was loaded into the reagent cartridge and run on MiSeq (Illumina, Inc., San Diego, CA, USA) for two ends by 150 bp reactions (Illumina) following manufacturer’s instructions.

### Metagenomic Shotgun Sequencing Protocol

DNA integrity was confirmed by gel electrophoresis and sent to Los Alamos National Laboratory to run on Illumina HiSeq platform. The DNA was fragmented and the library was prepared using TruSeq Kit (Illumina) according to manufacturer’s protocol. Each of the 19 samples was sequenced in one flow cell lane with 2 × 150 bp paired-end format.

### Paired-End Sequence Merging and Quality Trimming

Reads were merged using PEAR (Zhang et al., 2014) (options: -p 0.001). Both merged and unmerged reads underwent quality trimming using the SolexaQA package (Cox et al., 2010); reads were trimmed where Phred quality scores dropped below 17.

### Use of Publically Available Metagenomes from Distant Tundra and Temperate Grassland and Agricultural Habitats

Publically available metagenomes that were used for comparison purposes represent the 10–20 cm and 50–60 cm depths from Nome Creek, AK (NC; 200 km from CiPEHR site; Taks et al., 2014), active layer soil (30–35 cm depth) from Bonanza Creek, AK (BC; 100 km from CiPEHR site; Hultman et al., 2015), Toolike Lake LTER study site (TL; 530 km from CiPEHR site; Fierer et al., 2012), temperate steppe ecosystem in inner Mongolia, China (ZXM; Zhang et al., 2016), and agricultural soil from Urbana, IL, USA (UIL; Orellana et al., 2014). All datasets were processed and analyzed as described above for CiPHER and KFFL datasets for consistency purposes, when possible. The Toolik Lake metagenome was omitted for comparisons of taxonomic composition and community complexity because the dataset size was comparatively smaller (<500 mbp). A summary of each site, including sampling depth, year of sampling, etc. is provided in **Supplementary Table S2**.

### 16S rRNA Analysis from Amplicon PCR and Shotgun-Metagenome Reads

Amplicon PCR 16S rRNA sequences longer than 190 bp (75% of expected length, 253 bp) after trimming were used for further analysis. QIIME was employed for the majority of downstream analysis (Caporaso et al., 2010). 16S rRNA gene (16S) amplicon sequences were assigned to the sample they came from using a unique 12 bp barcode identifier, allowing for up to one mismatch. The Python script pick_de_novo_otus.py was used to cluster 16S sequences at 97% similarity (97% OTUs) with UCLUST (Edgar, 2010). Representative sequences of each OTU were taxonomically identified with the RDP Classifier (Wang et al., 2007). Representative sequences were also aligned using PyNAST (Caporaso et al., 2010), and a phylogenetic tree was constructed from this alignment using FastTree with default settings (Price et al., 2010). Information on dataset quality and number of sequences used per sample is available in **Supplementary Table S3**.

The relative abundances of various prokaryotic taxa were also determined based on 16S sequences recovered in the metagenomes. Metagenomic reads were trimmed and sister reads were merged using the same approach as above. 16S sequences were identified by searching all merged and unmerged reads longer than 70 bp after trimming against the May 2013 release of Greengenes 16S ribosomal database (DeSantis et al., 2006) pre-clustered at 97% identity, using blastn (BLAST + version 2.2.29, options: -word_size 16 -outfmt 6 -task blastn -dust no -max_target_seqs 1) (Camacho et al., 2009). Only matches along the V4 region of the 16S sequence with bit-score ≥60, e-value<1E-7, and match length ≥70 bp were retained for analysis. 16S sequences then underwent open-reference OTU-picking against Greengenes database pre-clustered at 97% (options: -m uclust_ref –s 0.97) (Edgar, 2010) (similar to methods performed in Luo et al., 2014a). Representative sequences of each OTU were taxonomically identified with the RDP Classifier (Wang et al., 2007). Taxonomy abundances for each sample were determined by the number of sequences that assigned to OTUs corresponding to that taxonomic group, divided by the total number of reads that passed open-reference OTU-picking step (i.e., that were used in clustering and not discarded). Euclidian distances of OTU tables were calculated, and the statistical significance of tundra vs. temperate topsoil OTU differences was determined by ANOSIM analysis. Results generalized at the phylum level were highly consistent between 16S rRNA gene analyses derived from 16S PCR amplicon sequences and 16S sequences recovered from metagenomic datasets.

### Analysis of Shotgun-Metagenome Short Reads

Protein prediction of metagenomic sequences ≥100 bp after trimming was performed with FragGeneScan (Rho et al., 2010) (Illumina 1% error model). Resulting amino acid sequences were searched against Swiss-Prot database [UniProt Consortium (2015)], using blastp (BLAST + version 2.2.28) (options: –word_size 3 outfmt 6, cutoff: bit score >75, alignment length ≥25 amino acids, amino acid identity ≥40%) (Camacho et al., 2009). Corresponding Gene Ontology (GO) annotations of functions and processes for each Swiss-Prot entry was obtained from http://www.uniprot.org/downloads (downloaded on December 04, 2014). Dataset quality and number of sequences used per sample is displayed in **Supplementary Table S4**.

A raw (not-normalized) counts table of genes and GO pathways (with sample metagenomes as columns and gene annotations or metabolic process categories as rows) was processed with the DESeq2 package (Love et al., 2014) to identify differentially abundant pathways between the two study sites and to generate log2 transformations of gene/process abundance ratios. The raw count data underwent a variance-stabilizing transformation, which is used for logarithmically distributed count data with low mean values that tend to have high variance. This transformation results in new values that have a relatively constant variance along the range of mean values and confers a reduced false positive rate for less abundant genes (Anders and Huber, 2010). *P*-values were transformed to account for false discovery rate from multiple testing using Benjamini–Hochberg correction (adjusted *p*-values; Benjamini and Hochberg, 1995). DESeq2 was also used to generate a sample-to-sample distance heatmap based on Euclidean distances derived from variance-stabilizing transformations of the raw count information, as well as to generate PCA plots based on the same transformations in order to visualize the overall effect of experimental covariates and batch effects.

### Assembly and Characterization of Population Bins

Initial assembly of metagenomic sequences was performed on each of the 11 CiPEHR Alaska datasets individually with CLC assembly package (CLC Assembly Cell 4.2.2)^1^ (options: -w 53). Resulting contigs ≥2 kbp were pooled together into one file and short metagenomic sequences were recruited to each contig to calculate the median coverage of each contig in each metagenome dataset (megablast with default; cut-off used: ≥90% of length of the query sequence, ≥98% nucleotide identity). Contigs ≥2 kbp were then binned using CONCOCT (Alneberg et al., 2014). Contigs that were not binned with CONCOCT were added to existing bins generated with CONCOCT if the unbinned contig matched a binned contig ≥99.7% nucleotide identity and ≥2 kbp alignment length (using megablast with default settings). For each bin, re-assembly was performed using only short reads matching the binned contigs; matches ≥98% nucleotide identity and ≥100 bp length were assembled *de novo* with Velvet (Zerbino and Birney, 2008), including both merged and non-merged paired-end reads, using odd-numbered word sizes between 85 and 99. These larger word sizes were chosen to increase assembly quality. Only contigs >1 kbp generated by this final assembly were included in the final bin; all contigs <1 kbp and reads and contigs from previous steps were discarded. CheckM was used to estimate completeness and contamination of each bin based on the recovery of 104 single copy universal bacterial genes (Parks et al., 2015).

Protein-coding genes of the assembled bins were predicted with MetaGeneMark (Zhu et al., 2010) and were searched against the Swiss-Prot database as described above [UniProt Consortium (2015)]. The sequence abundance of each bin was determined by the number of merged reads ≥200 bp from each metagenome that matched a population bin genome at ≥200 bp match length and ≥98% nucleotide identity. Closest ancestry was determined for certain organisms containing full or partial 16S and 23S rRNA sequences by searching contigs against SILVA small subunit (SSU) and large subunit (LSU) databases (v119 releases) (Quast et al., 2013) using megablast. A tree of phylogenetic relatedness based on 23S sequences was made for *Acidobacteria* bins 03, 06, 07, and 12 using *Ca. OP8* as an out-group, including several *Acidobacteria* isolates and uncultured organisms. Fasta sequences were aligned using MUSCLE algorithm (Edgar, 2004) and PyNAST for tree building (Caporaso et al., 2010). The resulting phylogenetic tree was visualized with FigTree V1.4.2^2^.

### Assessing Intra-Population Diversity and Biogeography

To assess whether the assembled bins were distributed over large geographical areas, trimmed reads from several publicly available metagenomes were searched against each population bin (reference) sequence using megablast. Matches ≥60% nucleotide identity and ≥70 bp length were retained. Fragment recruitment of these matches was performed to assess evenness and percent representation of each population bin in the corresponding metagenomes. Population structure was assessed using recruitment plots, essentially as described previously for marine metagenomes (Konstantinidis and DeLong, 2008) and below.

To further validate the results of the recruitment plots, the full-length (3,211–4,462-bp) β subunit of the bacterial RNA polymerase (*rpoB*) gene for the population/bin of interest was used to recruit highly matching sequences from the metagenomes of the same or different sites. *rpoB*-encoding reads were identified using megablast. All matching reads were then used in a Velvet assembly to reconstruct partial or full-length sequences, when possible (e.g., high enough coverage). Assembled *rpoB* sequences were aligned and then truncated to retain only the overlapping regions of all assembled *rpoB* sequences (overlapping region of CiPEHR original assembly and Nome Creek partial or full *de novo* assembly). Using megablast, unassembled metagenomic reads matching the *rpoB* sequences in the alignment were identified from CiPEHR, Nome Creek, and Toolik Lake metagenomes, using as cut-off for a match ≥85 bp alignment and ≥98% nucleotide identity. Representative reads from each location were added to each existing alignment with MAFFT Multiple Sequence Alignment (options: mafft –anysymbol –addfragments –multipair) (Katoh and Frith, 2012), and a tree was generated using Randomized Axelerated Maximum Likelihood, or RAxML, version 730 (Stamatakis, 2006). The resulting tree was visually inspected for the existence of a star-like phylogeny, indicating no population structure.

We developed a resampling approach to identify genes absent from *in-situ* populations in distant tundra habitats (NC, BC, or TL) that otherwise closely matched (>95% Average Nucleotide Identity [or ANI; Konstantinidis and Tiedje, 2005], >90% genome representation) one of the 27 reference CiPEHR population bin assemblies. Population bin genomes were broken up into 500 bp segments *in silico* and the average coverage for each segment was calculated independently, using recruitment of all available unassembled short sequences from the target metagenome. A skewed normal distribution was fit to these coverage values using the ‘enveomics.R’ package available for download at http://enveomics.blogspot.com. The parameters of the resulting skewed distribution were used to calculate the probability that a gene with zero or near-zero coverage (i.e., no or few reads matching to that gene) could occur by chance (null hypothesis being that the gene was present in the population). 500 bp segments were treated as independent tests, and thus, *p*-values for each gene were adjusted by their corresponding lengths as follows: *p*-value ^∧^ ([gene length]/500 bp). Therefore, comparatively longer genes were more robustly assessed for presence/absence. This analysis was performed on all genes for selected comparisons, and *p*-values were adjusted to account for false discovery rate from multiple testing using Benjamini–Hochberg correction (Benjamini and Hochberg, 1995)

### Data Availability

Assembled population bins, as well as raw shotgun metagenome and 16S rRNA gene sequences from CiPEHR, AK and KFFL, OK, are deposited in the European Nucleotide Archive^3^ under study no. PRJEB10725.

## Results

### Relative Microbial Community Complexity of Tundra Soils

**FIGURE 1:**
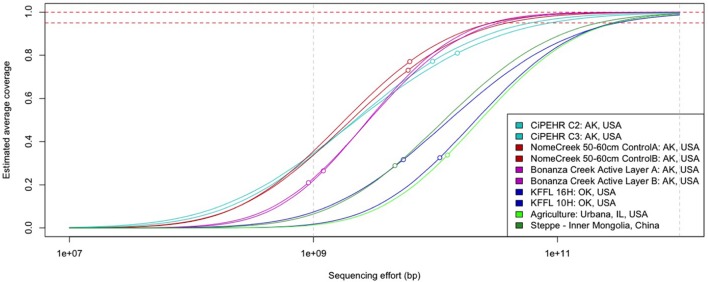
**Curves representing soil microbial community complexity estimations as determined by Nonpareil.** Nonpareil is a statistical tool that uses read redundancy to estimate dataset complexity and the amount of sequencing effort needed to achieve a desired level of coverage. Circles on curves represent the coverage of the actual sequencing depth for each dataset in relation to the entire curve (projection for complete coverage after the circle). Curves positioned on the right represent more sequence diverse metagenomes than curves positioned on the left.

Using Nonpareil, a statistical tool that employs read redundancy to estimate the coverage of the microbial community achieved by a metagenomic dataset (Rodriguez-R and Konstantinidis, 2014), a much more diverse community was observed in temperate soils compared to those from Alaskan tundra. The estimated sequencing depth required to sample 95% of the total extracted community DNA for each CiPEHR, AK soil sample was found to be 53.6 ± 5.45 Gbp (mean ± one standard deviation), 56.0 ± 25.7 Gbp for Nome Creek 10–20 cm and 50–60 cm depth metagenomes, and 34.6 ± 14.7 Gbp for Bonanza Creek active layer (∼30–35 cm depth) metagenomes (**Figure 1**; **Supplementary Table S5**). Using temperate soil metagenomes for contrast, an estimated 450 ± 15.2 Gbp of sequencing depth would be required to sample 95% of sequencing richness for Kessler Farm Field Laboratory site in Oklahoma (KFFL, “OK” site) soils, 281 Gbp for Urbana, IL (UIL) agricultural soil, and 215 Gbp for temperate steppe soil from inner Mongolia, China (ZXM). Based on a total of 139 Gbp and 62.2 Gbp available from all replicated metagenomes from CiPEHR and KFFL (merged sequences only), an estimated 91.8% of the combined ‘site community’ for CiPEHR and 62.2% for KFFL was sampled. Notably, the ‘site community’ represented by all 11 combined CiPEHR metagenomes *still* possessed less than half the complexity (or estimated sequence richness) estimated from a single KFFL metagenome. A higher level of diversity in the OK soil microbial community is further reflected in the 97% OTU rarefaction curve from 16S PCR amplicon sequences, where the number of OTUs detected at OK is over twofold greater than the number of OTUs observed at AK (**Supplementary Figure S1**).

### Microbial Community Compositional Differences between Study Sites

**FIGURE 2:**
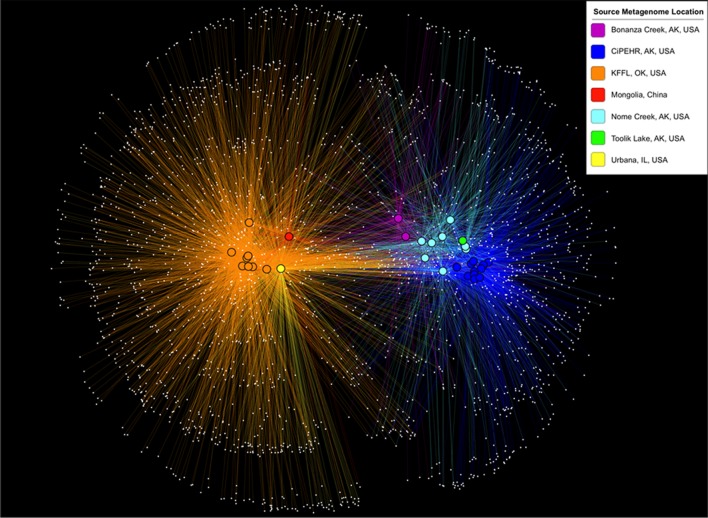
**OTU sharing network based on 16S rRNA gene sequences from metagenomes.** Samples are clustered (positioned) according to the presence and abundance of their shared OTUs (using make_otu_network.py, a QIIME script; Caporaso et al., 2010). White dots represent OTUs, and a line connecting these dots to a bolded sample dot indicates that the OTU is present in that sample. The colors of lines and bolded sample dots correspond to the source location of each metagenome as provided in the key.

Using 16S PCR amplicon sequences prepared in parallel from KFFL, OK and CiPEHR, AK soil samples, most OTUs were shared between soil samples from the same site, but comparatively few OTUs were shared between the two sites. In particular, <6% of OTUs were shared (*n* = 766, from a total of 8,290 non-singleton OTUs present at OK and 6,292 at AK) and these differences were statistically significant (*p*-value < 0.001; ANOSIM) (**Supplementary Figure S2**). Further, by using metagenome-extracted 16S sequences to compare CiPEHR and KFFL datasets with those publically available, it was found that, on average, 72.5% of the OTUs from active layer Bonanza Creek and 74% of the OTUs from Nome Creek soils were detectable in other tundra locations (**Figure 2**). These results revealed that a high degree of OTUs are shared within the Alaskan tundra ecosystem vs. between ecosystem types (*p*-value < 0.01; ANOSIM), regardless of slight methodological differences between studies (DNA extraction procedure, handling, etc.). This trend is not observed when OTU affiliations are summarized into broad taxonomic groups, such as phylum-level evaluation (*p*-value > 0.1; ANOSIM).

16S fragments recovered from metagenomes revealed several broad taxonomic groups that were common to either tundra or temperate ecosystems (i.e., ubiquitous in one, absent from the other). Namely, methanogenic archaeal classes *Methanomicrobia and Methanobacteria* were present in all tundra locations (CiPEHR, BC, and NC) (0.22% of total community, on average), but were non-detectable in any temperate soil metagenome (KFFL, ZXM, and UIL). Other groups specific to tundra soils include *Chlorobi* class *Ignavibacteria*, *Bacteroidetes* class *Bacteroidia*, *Crenarchaeota* class *MCG*, *Nitrospirae* family *Thermodesulfovibrio* (obligate anaerobic sulfate reducer; Henry et al., 1994; Sekiguchi et al., 2008), *Chloroflexi* class *Dehalococcoides*, and phylum *Lentisparae*. Conversely, *Nitrososphaeraceae*, an archaeal family of ammonia oxidizers, was detected in all temperate soils (∼1% abundance, on average), but was non-detectable in all tundra soils. *Nitrosomonas*, an ammonia oxidizing bacterium (Campbell et al., 2011), displayed patchy representation in OK 16S rRNA amplicon datasets, but was non-detectable in all tundra metagenomes or when using CiPEHR 16S rRNA amplicon sequences (**Supplementary Table S6**).

PCR amplicon sequences of the 16S rRNA gene (which allowed for a greater number of sequences per sample for analysis, relative to using 16S sequences derived from metagenomes) revealed that phylum *Acidobacteria* dominated the CiPEHR soil communities, representing 51.2% ± 11.62% of all members (mean ± one standard deviation; **Supplementary Table S7**), followed by *Proteobacteria* (15.5% ± 6.2%), *Verrucomicrobia* (15.1% ± 4.4%), *Actinobacteria* (8.7% ± 2.8%), and *Bacteroidetes* (2.7% ± 4.8%). Oklahoma soils were instead dominated by *Proteobacteria* (29.2% ± 4.4%), *Verrucomicrobia* (20.9% ± 6.6%), *Acidobacteria* (20.2% ± 1.9%), *Actinobacteria* (9.2% ± 2.8%), *Bacteroidetes* (4.0% ± 1.0%), *Planctomycetes* (3.3% ± 1.2%), and *Firmicutes* (2.5% ± 1.7%). Less-dominant phyla that were markedly more abundant at OK *vs.* AK were *Cyanobacteria* (0.72% *vs*. 0.10%; OK mean *vs*. AK mean), *Chloroflexi* (1.80% *vs*. 0.41%), *Nitrospirae* (1.21% *vs*. 0.24%), *Crenarchaeota* (0.37% *vs*. 0.02%), *OP3* (0.13% *vs*. 0%), and TM6 (0.15% *vs*. 0.01%). AD3 (1.01% *vs*. 0.29%; AK mean *vs*. OK mean), and *Chlorobi* (0.48% *vs*. 0.04%) represented low abundant phyla that had a markedly greater mean abundance at CiPEHR compared to KFFL.

### Metabolic Comparison of Tundra and Temperate Soils

Initial benchmarking with different read lengths indicated that a comparison including publically available metagenomes would not be robust given that sequences of different lengths presented inconsistent annotations; a finding consistent with those reported by Luo et al. (2014b). The publically available DNA sequences were all shorter than the 2 × 150 bp sequences available from CiPEHR, AK and KFFL, OK soil metagenomes. Thus, functional gene comparisons were limited to the CiPEHR, AK and KFFL, OK datasets.

Gene content dissimilarity derived from Euclidean distances of variance-stabilized data in DESeq2 (**Supplementary Figure S3**), displays high contrast between the CiPEHR, AK and KFFL, OK sites relative to dissimilarity between samples within a site. Analysis of functional genes from metagenomes involving the degradation of SOM revealed site-specific patterns for several processes. SOM degradation genes that were significantly more abundant in AK soil metagenomes compared to OK included those involved in the catabolism of chitin (79.3% percent higher abundance in AK relative to OK), cellulose (73.2%), simple carbohydrates (81.2%, on average, for monosaccharides; 40.1%, on average, for sugar acids; 68.6%, on average, for sugar alcohols), and lipids such as triglycerides and phospholipids (80.6 and 62.9%, respectively) (adjusted *p*-value <1e-3; Negative binomial test with DESeq2; **Supplementary Figure S4**). These findings were also congruent with previous studies of Artic tundra soils that found high numbers of chitinase, sugar alcohol, and mono- and disaccharide degradation genes in metagenomes (Yergeau et al., 2010) and metatranscriptomes (Tveit et al., 2014). Genes related to the catabolism of lignin and phenolic compounds were 71.0 and 58.9% more abundant in OK metagenomes, respectively (adjusted *p*-value <1e-3; DESeq2; **Supplementary Figure S5**). Genes corresponding to the Wood–Ljungdahl carbon fixation pathway (carbon monoxide dehydrogenase/acetyl-CoA synthase subunit alpha and 5-methyltetrahydrofolate:corrinoid/iron-sulfur protein co-methyltransferase proteins), which are primarily used by acetate-producing bacteria and methanogens (Roberts et al., 1994; Matschiavelli et al., 2012), were 50–200 times more abundant in AK metagenomes, relative to OK (**Supplementary Table S8**).

While genes related to denitrification (N-loss from ecosystem) were more abundant at OK (e.g., >3 times greater abundance of *nosZ* at OK), genes related to N-fixation (N-gain) were significantly more abundant in AK relative to OK datasets (46%, on average) (adjusted *p*-value < 1e-3; DESeq2; **Supplementary Figure S5**). More specifically, NifH nitrogenase iron protein was more than 80 times more abundant in AK relative to OK metagenomes (**Supplementary Table S8**). SoxAX cytochrome complex subunit A, a protein responsible for sulfur oxidation, was much higher in abundance in OK metagenomes, whereas genes involved in sulfate reduction (primarily sulfate adenylyltransferase subunit proteins), hydrogen sulfide biogenesis and fermentation genes were more abundant in AK soils.

### Contig Binning and Population Reconstruction from Alaska Soil Metagenomes

Contig binning resulted in the recovery of 27 population bins, 12 of which were >80% complete based on the recovery of 104 single-copy universal bacterial genes using CheckM (Parks et al., 2015), with 0–1.7% contamination (**Table 1**; **Supplementary Table S9**). Fourteen of the bins had an N50 (i.e., the length that >50% of the assembly is in contigs of this length or longer) between 20,203 bp and 218,163 bp. Collectively, the 27 assembled population bins recruited 7.5% of all reads, on average (9.2% max), for the 11 metagenomes (≥98% nucleotide identity; ≥200 bp alignment).

**Table 1:** Summary statistics for the most complete population bins.

	Population bin contigs				Single copy genes			CheckM results	
			
Bin ID	n50 (bp)	Longest (bp)	Total Length (bp)	Number Contigs	0	1	2	Completeness %	Contamination %
1	87585	258416	4341484	85	5	98	1	94.0	1.7



2	44196	144885	4793253	228	21	83	0	89.7	0.0
3	167654	415574	5822639	110	4	100	0	95.7	0.0
4	36041	146424	2468837	156	14	90	0	81.0	0.0



5	20203	76412	2053920	189	12	92	0	83.5	0.0



6	54187	226195	3684989	154	2	102	0	97.4	0.0



7	142525	388009	5910590	149	12	92	0	89.8	0.0
8	65109	167976	3268372	73	3	100	1	95.7	1.7



10	12295	50046	2301754	289	18	85	1	81.3	1.7
11	51987	197629	5172412	191	2	102	0	98.8	0.0
20	13258	53656	3231835	375	31	71	2	83.1	1.0
22	27045	163169	4115219	235	30	74	0	74.4	0.0%
27	26244	147429	8218361	566	3	101	0	94.8	0.0


*Assembly statistics, including N50 (i.e., the length that >50% of the assembly is in contigs of this length or longer), the length of the longest contig, the total length of all contigs combined, and the number of contigs comprising a bin, as shown. All contigs were >1 kbp long. CheckM was used to assess each bin for completeness, evaluating the presence of 104 single copy bacterial genes. The number in each column represents the number of instances each of the 104 bacterial single copy genes was found in each bin (i.e., 98 single copy genes were found once in Bin 01). Bins highlighted in green indicate those detected in distant tundra locations (100–530 km from CiPEHR, AK) at >2X representation of the genome. Single copy marker genes that occur more than once served as an indicator of contamination – i.e., that the assembly is combined with sequences from a more than one organism.*

**FIGURE 3:**
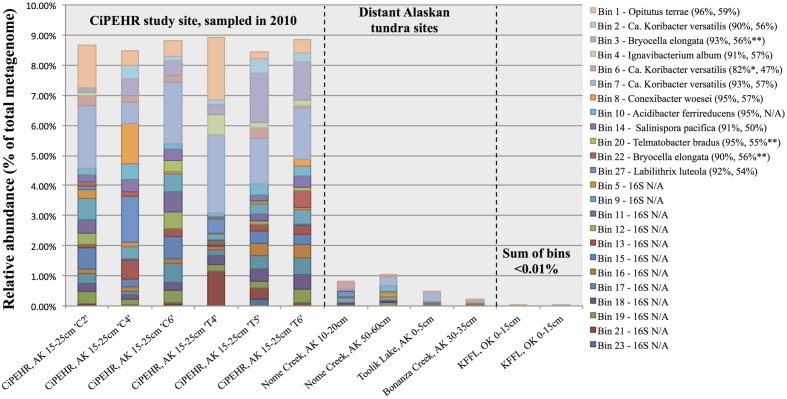
**Abundance of individual populations (bins) in various Alaskan tundra metagenomes determined by read mapping.** Results were obtained by the number of sequences that matched a population genome at ≥200 bp and ≥98% nucleotide identity for CiPEHR metagenomes (≥90 bp and ≥98% nucleotide identity for metagenomes at other locations), divided by the number of reads used as query (all merge-able paired-end sequences ≥200 bp at CiPEHR; sequences ≥90 bp at other sites). Bin abundance is given for two KFFL, Oklahoma metagenomes for contrast. Taxonomic affiliation is provided for bins with full or partial 16S ribosomal sequence, along with the average amino acid identity (AAI) to the corresponding genome. ^∗^Nucletide identity given corresponds to the 23S sequence because the 16S gene was not available. ^∗∗^AAI is derived from comparison to a genome that is not the same as the 16S match because a genome sequence was not available for these references; instead, the next closest match was used.

**FIGURE 4:**
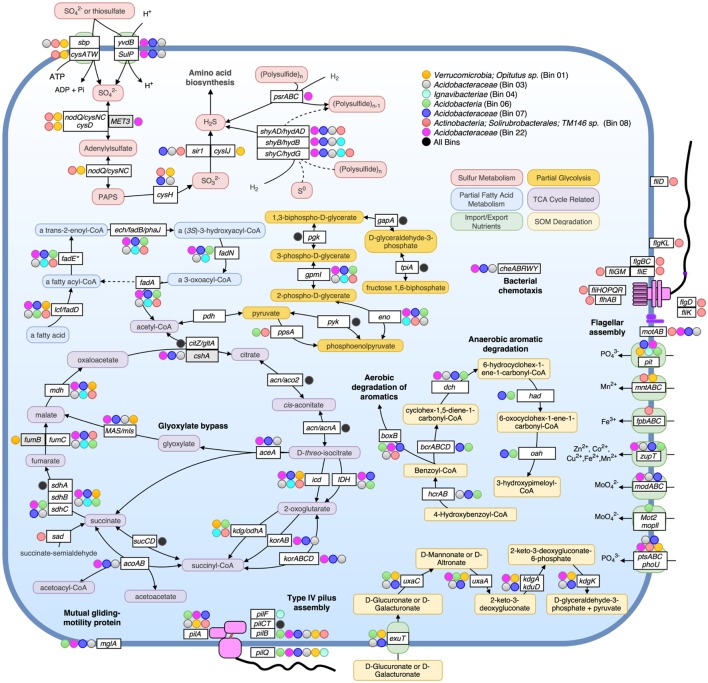
**Metabolic pathways identified in seven assembled population bins.** Selected metabolic transformations and other cellular activities are represented by both the name of the gene known to encode the protein enzyme for them as well as the substrate of the enzyme. Protein enzymes were identified by searching predicted proteins against Swiss-Prot database, as well as by searching against close representatives of selected organisms: *Acidobacteria Ca. Solibacter usitatus Ellin6076* and *Ca. Koribacter versatilis Ellin345* for bins 03, 06, 07, and 22; *Chlorobi Ignavibacterium album JCM 16511* for bin 04; *Verrucomicrobium Opitutus terrae PB90-1* for Bin 01. In all instances besides *zupT*, *mglA*, *phoU*, *sad*, and *pit*, pathways were only displayed if all or most genes of an operon involved in the same pathway/process were detected as present. Colored circles alongside genes indicate that the bin assigned to that color (see legend on top right) encoded the gene (blastp cutoffs: *e*-value < 1e-20, bitscore > 100). A black-filled circle indicates that all seven bins in the upper right box possessed the accompanying gene.

We have chosen to highlight seven of the most complete population bins below (see also **Table 1**; **Figures 3** and **4**). Bin 01 contained full-length 16S and 23S rRNA gene sequences matching at 96 and 93.1% nucleotide identity, respectively, to an *Opitutus sp.* (phylum *Verrucomicrobia*) isolate from an anoxic region of rice paddy soil (Chin et al., 2001) (**Supplementary Table S10**). Protein annotation of this bin revealed the presence of genes related to methanotrophy, including methylamine and methanol pathways (*mtaB, mtbA, mttC1*). Bin 01 also contained genes involved in assimilatory sulfate reduction and transport (*sbp*, *cysATW, cysDGHIJK, nodQ*), the degradation of a variety of organic compounds, including L-arabinose, xylan/xyloglucan, D-glucuronate, ribose, cellulose, and rhamnose, and the metabolism of molecular H_2_ (*hndACD, hoxHY, hypABCDEF, hupE*).

Bins 03, 07, 11, 12, and 22 were closely related (>80% ANI) and together formed a novel monophyletic clade within the family *Acidobacteraceae* based on partial 23S rRNA gene sequences (**Supplementary Figure S6**). Bin 03 contained 100/104 single-copy bacterial genes, and partial 16S and 23S rRNA gene sequences (338 and 568 bp, respectively; **Supplementary Table S10**). The 23S sequence matched family *Acidobacteriaceae* (94.4% nucleotide identity). Bin 07, possessed a partial (358 bp) 23S sequence that matched *Granulicella mallensis MP5ACTX8* (also *Acidobacteriaceae*) at 94.7% nucleotide identity, which was previously isolated from tundra soil in Finland (Männistö et al., 2012). Bin 03 and 07 shared many functional genes; including Citric Acid Cycle (TCA) with glyoxylate bypass (*icl*, *MAS*), in addition to genes required for ‘typical prokaryotic TCA metabolism.’ Both bins possessed genes for trimethylamine utilization and degradation (*mttBC*, *etfABD*, *mauG*) and an ammonium ion transporter on the same operon (*nrgA*), and several genes involved in the degradation of many SOM constituents, such as rhamnose import and catabolism (*rhaT, rhaB, rhaM*), degradation of fatty acids (*lcf*, *fadADEN*, *ech*), genes for catabolism of succinate (*sdhABC*), formate (*fdoGI*, *fdhE*, *fdnHG*), and several other genes for the catabolism of xylan, xyloglucan, cellulose, arabinan, and chitin and its derivative, *N*-acetylglucosamine. Bin 03 also contained genes for coenzyme B–coenzyme M heterodisulfide reductase (*hdrABC*) and both bins contained genes for other methane-related coenzyme activity (*cofDEGH*). Bin 07 possessed genes for anaerobic and aerobic degradation of aromatic compounds (*hcrAB*, *bcrABCD*, *dch*, *had*, *oah*, *boxB*). On average, 1.95% of metagenomic reads matched bin 07 at ≥98% nucleotide identity, making it the most abundant of the assembled populations at the AK site (**Figure 3**).

Bin 22 shared many metabolic strategies with bins 03 and 07. It also contained genes for TCA cycle with glyoxylate bypass (*icl*, *MAS*), as well as genes for the degradation of carbohydrates and fatty acids. All three bins possessed genes for bacterial chemotaxis (*cheABRWY*), motility proteins (MotAB), and along with bin 06, possessed mutual gliding protein (MglA), but none possessed genes necessary for flagellar assembly.

Bin 06 possessed 102/104 single-copy genes, and contained a large contig ending in a partial 23S ribosomal sequence matching a representative of “*Candidate Division OP8*” and an uncultured *Acidobacteria* at 79.9 and 80.2% nucleotide identity, respectively (**Supplementary Table S10**). Therefore, it probably represents a group more distant to known organisms than other assembled bins (**Supplementary Figure S6**). This bin contained genes for rhamnose, cellulose, and D-glucarate catabolism, hydrogen metabolism (*hyfBDEF*), sulfoxide reductase (*yedYZ*), and a wide variety of genes for the degradation of aromatic compounds (*hcrAB*, *bcrABCD*, *dch*, *had*, *oah*, *boxBC*, *nicAB*, *catIJ*, *mphP*).

Bin 04 possessed a partial 23S rRNA gene sequence (760 bp) matching to *Ignavibacteria* (phylum *Chlorobi*), and also contained Coenzyme B–Coenzyme M heterodisulfide reductase genes (*hdrABC*), which can be involved in anaerobic methanotrophy, sulfur reduction, or fermentative pathways. Bin 04 possessed only the beta and gamma subunits of the sulfyhydrogenase I complex (*hydGB*) implying that it likely transforms elemental sulfur into hydrogen sulfide with molecular H_2_, or performs the reverse reaction. Bin 08, which contained full 16S and 23 rRNA gene sequences, best matching actinobacterial family *Solirubrobacterales* (99.1 and 93.4% nucleotide identity, respectively), comprised of 73 contigs and contained 101/104 single copy genes. This genome possessed motility proteins (MotAB), as well as genes required for flagella assembly (*fliEGHKMOPQR*, *flhAB*, *flgBCL*). It also encoded genes for sulfate binding, import, and assimilation (*sbp*, *cysATW*, *nodQ*, *cysH*, *sir1*), polysulfide reduction using molecular H_2_ (*shyACD*), trimethylamine dehydrogenase (*tmd*), and genes related to the catabolism of fatty acids, chitin, oligopeptides, lipopolysaccharides, mannose, galactose, acetate, succinate and pyruvate.

None of the 27 bins possessed denitrification genes *narG, nirS*, *norB*, or *nosZ*. Bin 20 was found to encode all genes necessary for nitrogen fixation (*nifABDEHJKNQSV1WXZ*). Bin 20 also contained a contig ending in a partial 16S ribosomal sequence matching an *Acidobacteria* representative at 100% nucleotide identity (169 bp match) and 19/21 ribosomal proteins best matched to *Acidobacteria Ca. Solibacter* or *Ca. Koribacter*.

### Population Distribution in Other High Latitude Soils

**FIGURE 5:**
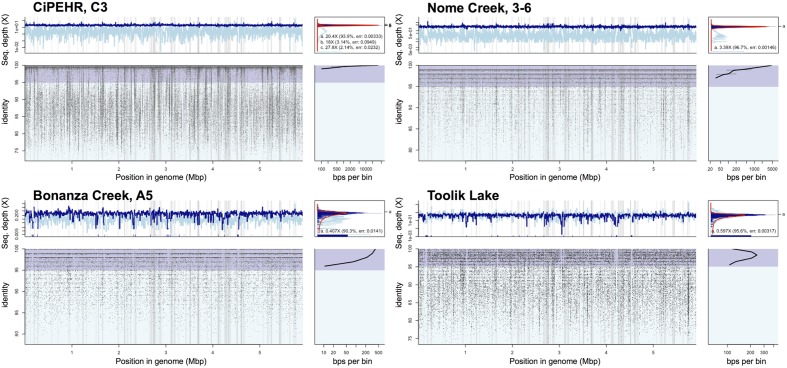
**Read recruitment plot of population bin 07 at four distinct tundra locations.** Diagrams expressing coverage of population bin 07 (assembled from CiPEHR, AK metagenomes) in metagenomic datasets from four distinct geographic locations, each separated by 100–530 km distances. All short reads from select sample metagenomes were searched against all contigs of the bin in a megablast search. The position histogram (top left of each) displays the average coverage of each base position, determined by a 1,000 bp window. The dark blue histogram represents the coverage at each base position determined from reads matching ≥70 bp in length and ≥95% nucleotide identity, while the light blue represents the coverage determined from reads matching ≥70 bp in length and <95% nucleotide identity. An even coverage across the entire contig from matching reads that are high identity (>95%) is indicative of a high quality contig from a single population. The recruitment plot (bottom left of each) shows where individual metagenomic reads matched to the population bin and the identity (%) of the match. The ID histogram (bottom right) displays the total number of short read-derived base-positions at given percent identities. Note that in all cases shown, a sequence-discrete population represented by reads showing high nucleotide identity to the reference population genome sequence (typically > 95% nucleotide identity) and even coverage across the length of the reference sequence is obvious.

Read recruitment plots against the assembled population bin genome sequences revealed that all bins represented sequence-discrete populations, with short reads matching population bin sequences mostly at 99–100% nucleotide identity, evenly across the entire genome (examples presented in **Supplementary Figure S7**). These findings revealed that soil microbial communities are composed of discrete populations, at least for the abundant fraction that is robustly assessed by metagenomics. These findings echoed results reported previously for other habitats (Caro-Quintero and Konstantinidis, 2012). Further, all 27 population bins were detected in more than one CiPEHR metagenome (**Figure 3**). Surprisingly, several bins matched reads in other publicly available tundra soil metagenomes (>98% nucleotide identity and even coverage across the genome sequence), indicating that these populations are widespread in high latitudes and revealing that long-lived, sequence-discrete, tractable populations are present in at least some soils. Specifically, datasets from the Nome Creek area (Taks et al., 2014), located ∼200 km Northeast from the CiPEHR site, contained five of the assembled bins at high abundance (i.e., 2-10X coverage of population bin with unassembled short sequences) (**Figures 3** and **5**; **Supplementary Figure S8**). Metagenomes containing these populations originated from surface (10–20 cm) and middle depth (50–60 cm) samples, which undergo seasonal thawing, similar to the CiPHER site. Metagenomes representative of the permafrost layer (90–100 cm) did not contain any of the assembled bins from CiPEHR. Bin 07, the most dominant population bin in CiPEHR and Nome Creek site metagenomes, was also found in active-layer soil from Bonanza Creek, AK (∼100 km Northeast from CiPEHR) and in a soil metagenome from Toolik Lake, AK (530 km North of the CiPEHR site; **Figure 5**), along with bins 09 and 10 (**Supplementary Figure S8**). Because fragment recruitment using Marcell Experimental Forest soil metagenomes (SPRUCE experiment in Northern Minnesota; Lin et al., 2014) revealed the presence of a distinct acidobacterial population closely related to bins 03, 07, and 11 (∼82% ANI), *de novo* assembly was performed on these metagenomes in order to recover the corresponding population bin(s) (**Supplementary Figure S9**).

### Genomic Adaptation to Fire Disturbance at Nome Creek, Alaska

Bins 07 and 09 were represented by ∼3X coverage or more in both fire-impacted (soil sampled 7 years following a fire disturbance event) and control (undisturbed) soil metagenomes from Nome Creek, AK (Taks et al., 2014). The adequate coverage of the CiPHER bins obtained in these samples, allowed for the investigation, at a fine resolution level, of the genomic adaptations likely occurring as a result of the fire-related environmental changes. Briefly, fire disturbance at this site resulted in a large reduction in SOM and soil moisture content, complete thawing of permafrost, and several years of elevated temperatures relative to adjacent undisturbed soils. All genes belonging to population bin 07 were represented in metagenome NC3-6 (50–60 cm depth, undisturbed; hence, complete representation of the population bin), with an average nucleotide identity of 99.6% using all recruited matches resulting from megablast (cutoffs: matching length >70 bp, nucleotide identity >60%) (**Figure 5C**). Comparatively, the average nucleotide identity of matching sequences in NC2-6 (50–60 cm depth, fire-impacted) was 99.1%, and 130 genes of the reference genome (2.5% of the total) were absent from the *in situ* population (adjusted *p*-values <0.025). Similarly, recruitment of short reads from metagenome NC3-6 revealed near-complete representation of population bin 09 genes with an estimated ANI of 99.5%, whereas 209 genes of the reference genome were absent from the NC2-6 *in situ* population, and an ANI of 98.9% estimated from the recruited sequences (adjusted *p*-values <0.025). Absent genes included those involved in transcriptional regulation (9 and 13% in bins 07 and 09, respectively), carbohydrate transport and metabolism (18 and 12%), amino acid transport and metabolism (13 and 14%), and nutrient transport (8 and 11%), among other functions and hypothetical genes. Examples of selected contigs from bins 07 and 09 with high gene absence in fire-impacted soils are displayed in **Supplementary Figure S10** (read recruitment from undisturbed soils are also shown for contrast).

## Discussion

### Assessment of Microbial Biogeography Reveals Conserved, Pervasive Bacterial Populations in the Alaskan Tundra Ecosystem

The high degree of microbial community heterogeneity often observed in soil systems remains poorly understood. Here, we reported the detection of discrete bacterial populations, several of which -but not all- were also found in locations that are separated by distances of up to 530 km (**Figures 3** and **5**; **Supplementary Figure S8**). These findings were consistent with previous studies that reported a large degree of shared genera between tundra locations separated by up to 70 km (Frank-Fahle et al., 2014). The patchy representation of certain population bins within and between study sites uncovers an inherent feature of this heterogeneity, i.e., although soils within close proximity (cm to m distances) may not share certain microbial populations, these populations can be shared by soil samples separated by several 100 km distances. These findings reveal that micro-variable soil conditions and niche space might be shared across large geographic regions in the tundra ecosystem, but not uniformly. The *Acidobacteria* bin 07, which possessed metabolisms for elemental hydrogen, elemental sulfur, sulfate, methanotrophy, TCA cycle with glyoxylate bypass, and catabolism of fatty acids, carbohydrates, and recalcitrant SOM such as chitin and aromatic compounds, was the most dominant population within the CiPEHR site metagenomes (**Figure 3**), and was also found, at high relative abundance (**Figure 5**), in metagenomes from three other tundra locations, all separated by ≥100 km. These findings revealed a successful generalist population, capable of thriving in many distinctive locations over broad geographical distances, and also suggests that microbial species conservation may dominate over high habitat specificity for certain soil prokaryotes. Furthermore, bins 03, 07, 11, 12, and 22 comprise a monophyletic group of closely related populations with >97% ribosomal gene sequence similarity (**Supplementary Figure S6**) or high ANI (∼80% or greater). A close relative to this group (82.2% ANI to bin 11; **Supplementary Figure S9**) was assembled directly from Marcell Experimental Forest soil metagenomes (∼3,780 km from CiPEHR; Lin et al., 2014), and recruited up to 6.8% of all metagenomic reads from its source metagenome. This finding underscores the high dominance of this relatively narrow (phylogenetic) *Acidobacteria* clade across large geographic distances (i.e., 3,780 km, or more).

Previous studies have demonstrated the rapid response of active layer tundra soil microbes to elevated temperatures in the laboratory (Mackelprang et al., 2011), and here, we demonstrated the tempo and mode of adaptation of tundra bacterial populations to environmental perturbations such as prolonged fire events. In this case, adaptation took place by altering 2–3% of the genome within a period of 7 years or less. This is quite remarkable for soils microbes, especially those in low-temperature high latitudes, which are traditionally viewed as slow growers (Price and Sowers, 2004). Many of the genes absent from *in situ* populations of fire-impacted soils (but present intact in undisturbed soils) were assignable to pathways related to the transport and catabolism of simple SOM substrates and gene regulation, implying that such disturbance events may have long lasting effects on soil C cycling. Taks et al. (2014) demonstrated that for middle layer depths (50–60 cm; same depth assessed in our own comparison) subject to fire, genes for simple carbohydrate metabolism decreased in abundance; here, we demonstrate that at least part of this change was attributable to the genomic alteration of existing, dominant microbial populations. Furthermore, the loss of transcriptional regulatory genes may reflect an alleviation of prior metabolic constraints (e.g., due to increased temperature or decreased moisture content in this case). Consistent with these findings, Coolen and Orsi (2015) demonstrated that transcriptional regulation and signal transduction represented a large category of genes that were differentially regulated between thawed or frozen permafrost soils. Our cross-site comparison exemplified the usefulness of combining datasets from multiple studies and showed that sequences and genomes recovered from soil can be meaningfully combined with datasets representing the same ecosystem elsewhere, at least in this Alaskan tundra ecosystem.

### Contrasting Taxonomic Composition and Functional Gene Content in Tundra and Temperate Topsoil

The assembled population bins were highly representative of both the taxonomic and functional composition of the whole-community in CiPEHR, AK soils, epitomizing several pathways that were more abundant at tundra relative to temperate metagenomes. Even though the bins were comprised of organisms from several phyla, many metabolic pathways were nearly ubiquitous amongst the bins. Namely, several organisms contained genes indicative of methane or methylamine metabolism(s). The majority of populations encoded genes for the formation or catabolism of gaseous hydrogen. Many also contained pathways for the degradation of SOM, capable of degrading compounds of varying recalcitrance, i.e., chitin, cellulose, cellobiose, xylan, mannan, xylose, β-galactosides, raffinose, arabinose, aromatics, etc., several of which are highly expressed or abundant in tundra soils, as found previously (Yergeau et al., 2010; Tveit et al., 2014). A large portion of the latter compounds is apparently plant-derived, revealing the tight coupling between aboveground plant and belowground microbial communities in the tundra ecosystem. While genes responsible for the degradation of more labile carbohydrates are in greater relative abundance in CiPEHR metagenomes, it is clear that genes related to the degradation of many recalcitrant compound categories, such as phenolics and lignin, are more abundant in KFFL, OK metagenomes compared to CiPEHR (**Supplementary Figures S4** and **S5**), consistent with the differences in aboveground plant communities between the two sites. Further, the presence of genes recognized as hydrogeno- and methano-trophic in annotated short reads and assembled population bins, along with the absence of archaeal population bins and low overall methanogen abundance determined by 16S rRNA community analysis, suggests that methane production likely occurs at deeper layers in CiPEHR sites and migrates upward toward the surface, where it is either released to the atmosphere or consumed by bacteria. Consistent with these interpretations, a recent study using metagenomes which included several of the assembled bins from CiPHER according to our analysis, displayed an overall reduced archaeal presence in active layer compared to permafrost layer soil depths (Taks et al., 2014).

Previous studies have shown that permafrost ecosystems are nitrogen-limited (Shaver and Chapin, 1980; Martineau et al., 2010; Wild et al., 2014) and this was reflected in both our own Illumina datasets as well as the 454 datasets determined by Yergeau et al. (2010) for permafrost soils. For instance, although evidence of nitrate/nitrite transport was present, none of the binned populations displayed a significant role in the denitrification process. It is possible that decreased denitrification potential (i.e., processes ultimately leading to nitrogen loss from the ecosystem) is a community adaptation to limiting N-availability, given that the genetic potential for this activity is lower at CiPEHR relative to KFFL, even though other metabolic pathways at CiPEHR are indicative of anaerobic conditions. N-limitation likely plays a role in constricting SOM degradation in permafrost ecosystems. If N-cycling in these systems was altered due to anthropogenic related changes to the ecosystem (e.g., agriculture practices and climate change), it is necessary to understand how this will relate to greenhouse gas emissions (climate feedbacks), i.e., an increase in N-availability would stimulate aboveground plant communities and act as a C-sink or would increase denitrification activity resulting in acceleration of N_2_O (a potent greenhouse gas) emissions. Furthermore, previous research demonstrated that addition of organic N to active layer tundra soil high in SOM content resulted in a 2-3-fold increase in SOM decomposition (Wild et al., 2014). Notably, one *Acidobacteria* (bin 20) possessed a complete assortment of nitrogen fixation genes, which is to the best of our knowledge, the first report of nitrogen fixation for a member of phylum *Acidobacteria*.

### Relative Taxonomic and Functional Complexity of Tundra and Temperate Soil Communities

Metagenomes representing temperate topsoils displayed greater taxonomic and functional diversity than those from Alaskan tundra. The lower diversity in tundra may be related to longer generation times, restrictive metabolic conditions (e.g., O_2_ restriction or lower temperature), and/or lower diversity of plant-derived organic carbon. Recent studies have shown that a constraint on diversity for certain functional traits results in decreased ecosystem functioning (Salles et al., 2012; Singh et al., 2014). Hence, changes in the diversity of functional genes relevant to SOM degradation may constrain or promote the rate at which these soils systems respond to environmental perturbations. Accordingly, the tundra microbial communities are presumably more vulnerable (less robust) to environmental change compared to their temperature counterparts.

### Summary and Future Work

The degree to which microbial variability differs between ecosystems is essential for research efforts endeavoring to holistically understand soil microbial ecology in the context of ecosystem functioning and how environmental changes affect microbial activities and services. Our analyses revealed several taxa, at different resolution levels, i.e., OTUs, taxonomic clades, or individual populations, that are ubiquitous, and frequently among the most abundant members of the corresponding communities, in the active layer of Alaskan tundra soils. Thus, their relative contribution to various ecosystem functions is expected to be high. These assembled bins and DNA sequences provided here represent potential model organisms for future *in situ* experimental manipulations; for instance, through the design of population-specific PCR for assessing gene transcript (activity) level, allowing potential linking of methane, nitrogen, SOM, etc. turnover to individual populations.

## Author Contributions

EJ was the Ph.D. student/research assistant leading the analysis and write up of this research project (i.e., first author). LR and CL provided direct assistance of bioinformatic analysis and interpretation for metagenomic and 16S data. MY prepared extracted DNA for sequencing on Illumina HiSeq and MiSeq platforms. LW, ZH, ES, and YL were involved in study site setup and upkeep, sample collection, and sample processing. ES, JT, JZ, and KK were involved in early organization and planning of the research project and in peer-editing of the manuscript, providing direct feedback to EJ during the write up of the project.

## Conflict of Interest Statement

The authors declare that the research was conducted in the absence of any commercial or financial relationships that could be construed as a potential conflict of interest.

## Footnotes

1 http://www.clcbio.com

2 http://tree.bio.ed.ac.uk/software/figtree/

3 http://www.ebi.ac.uk/ena/
