# Supplementary material for: Metagenomics Reveals Pervasive Bacterial Populations and Reduced Community Diversity across the Alaska Tundra Ecosystem
Source: Front Microbiol. 2016 Apr 25;7:579. doi: 10.3389/fmicb.2016.00579 (PMC4842900; doi:10.3389/fmicb.2016.00579)
Supplement: Supplementary file 1 [file Data_Sheet_1.DOCX]

**SUPPLEMENTAL FIGURES AND TABLES**


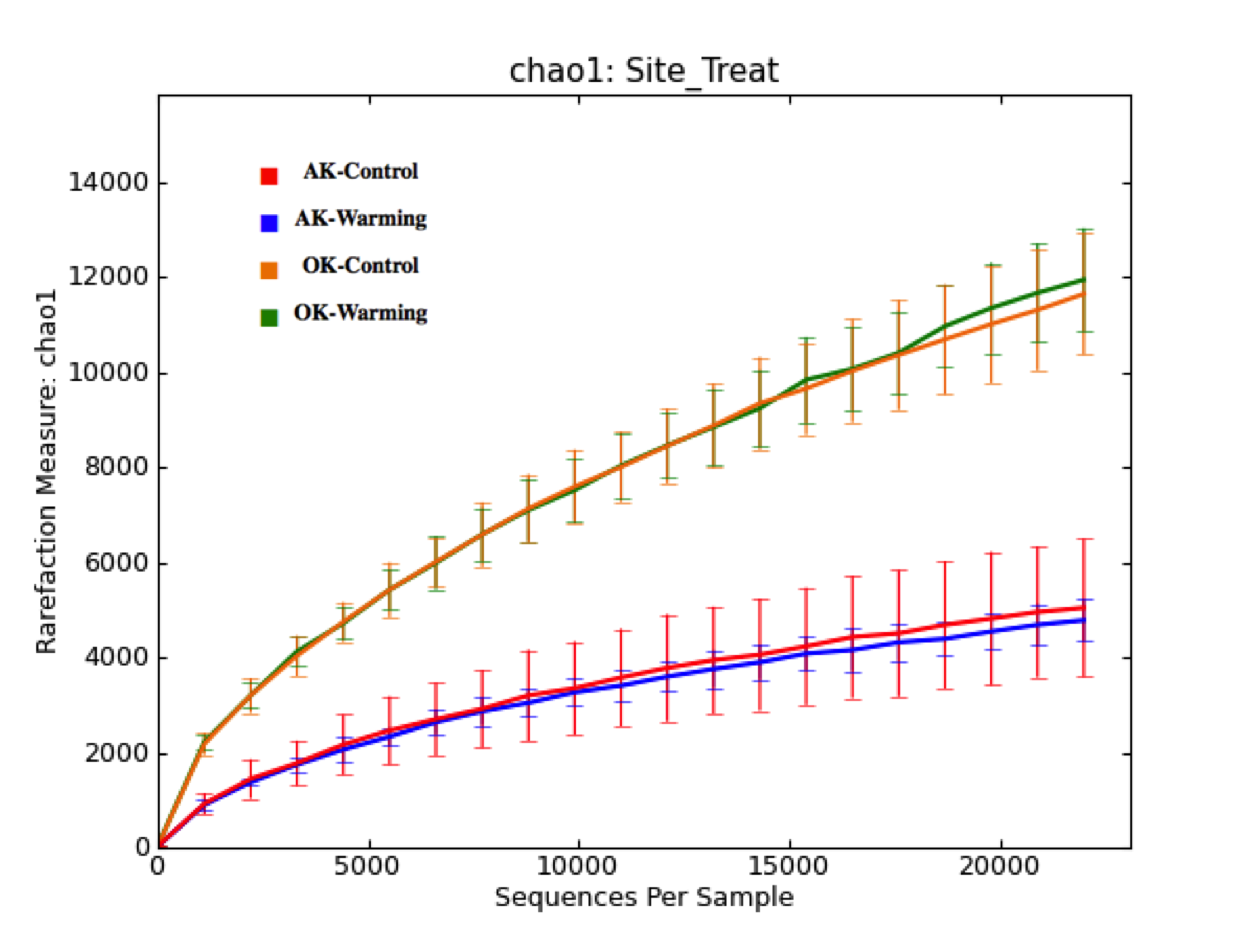


**Supplementary Figure 1 | OTU rarefaction curve.** Displays the number of OTUs obtained by subsampling at different sequencing depths; an estimate of microbial community diversity. Estimates are obtained by random sampling of a supplied OTU table of 16S rDNA gene amplicons from region V4 clustered at 97% similarity, with removal of singleton OTUs. Chao1 metric was used for alpha diversity measurement.

**Supplemental Figure 2 | OTU sharing network representing OTUs (PCR amplicons from 16S rRNA gene clustered at 97% similarity).** Samples are clustered (positioned) according to the presence and abundance of their shared OTUs (using make_otu_network.py, a QIIME script; Caporaso et al. 2010b). Bold red and orange dots represent Oklahoma soil samples, warming and control plots, respectively; Bold dark blue and light blue dots represent Alaska soil samples, warming and control, respectively. White dots represent OTUs, and a line connecting these dots to a bolded sample dot indicates that the OTU is present in that sample. After removal of singleton OTUs from the dataset (OTUs appearing only once), 8,290 OTUs are represented at the OK site and 6,292 OTUs are represented at the AK site, with only 766 OTUs shared between the two sites.


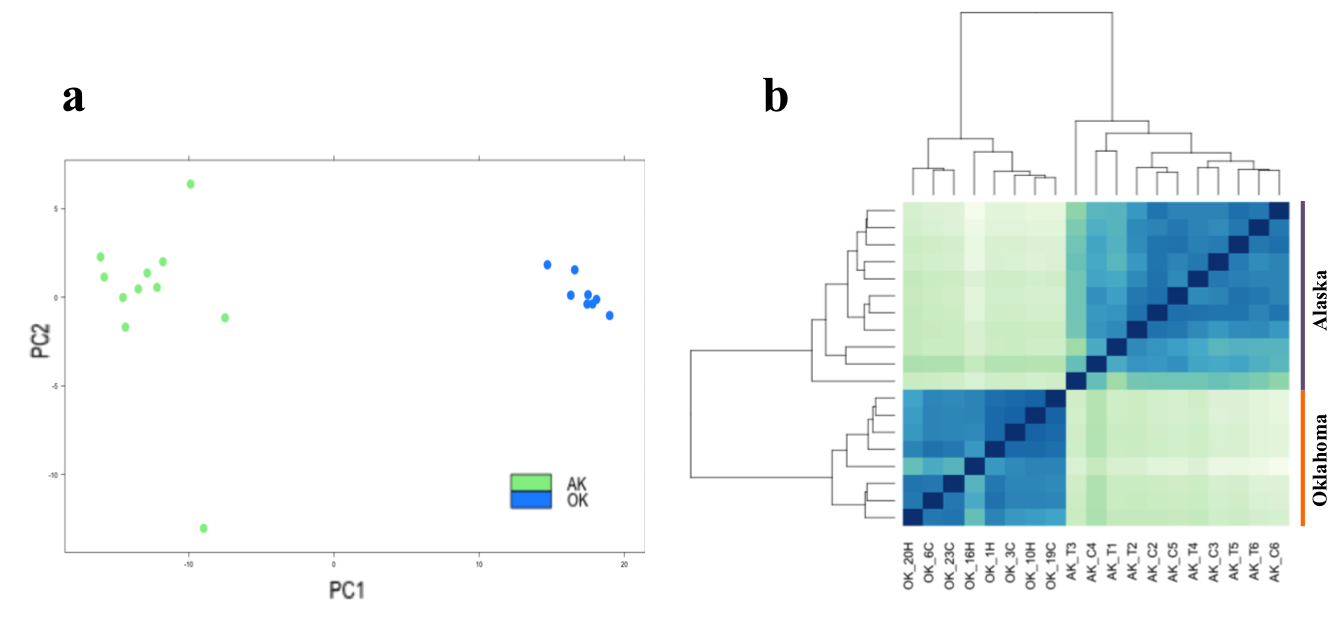


**Supplementary Figure 3 |** (**a**) **PCA plot.** The 19 samples shown in the 2D plane spanned by their first two principal components. Underlying data are gene count matrix (short read annotation to Swiss-Prot) which have undergone variance stabilizing transformation in DESeq2 package. (**b**) **Sample-to-sample distances**. Heatmap showing the Euclidean distances between samples as calculated from the variance stabilizing transformation in DESeq2.

**Supplementary Figure 4 | Log2-fold differences in gene abundance between OK and AK soil samples of select SOM catabolic pathways.** Dark grey bars represent pathways that were more abundant in AK metagenomes and light grey bars represent pathways that were more abundant in OK metagenomes. Pathways include (a) disaccharides, polysaccharides, cell wall constituents, hemicelluloses, cellulose, lignin, and chitin, a well as (b) sugar alcohols, sugar acids, and monosaccharides. SOM constituent catabolic pathways preceded with an asterisk denote those that were significantly different between sites (p-value < 0.001, DESeq2).


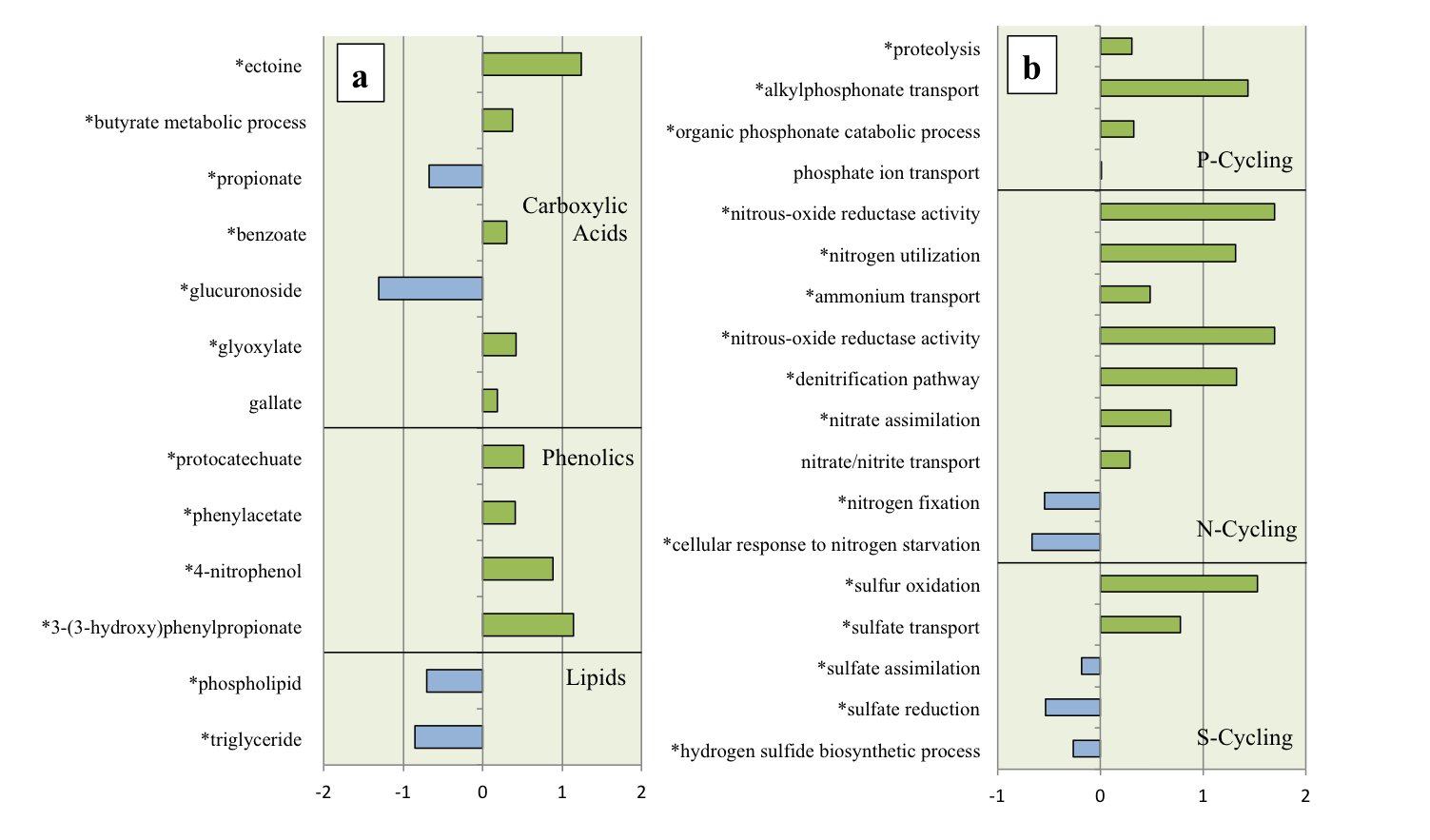


**Supplementary Figure 5 | Log2-fold differences in gene abundance between OK and AK soil samples for (a) catabolic pathways of lipids, phenolic compounds, and carboxylic acids, as well as (b) select S, N, and P-cycle pathways.** Blue bars represent pathways that were more abundant in AK metagenomes and green bars represent pathways that were more abundant in OK metagenomes. Pathways names preceded with an asterisk denote those that were significantly different between sites (p-value < 0.001, DESeq2).


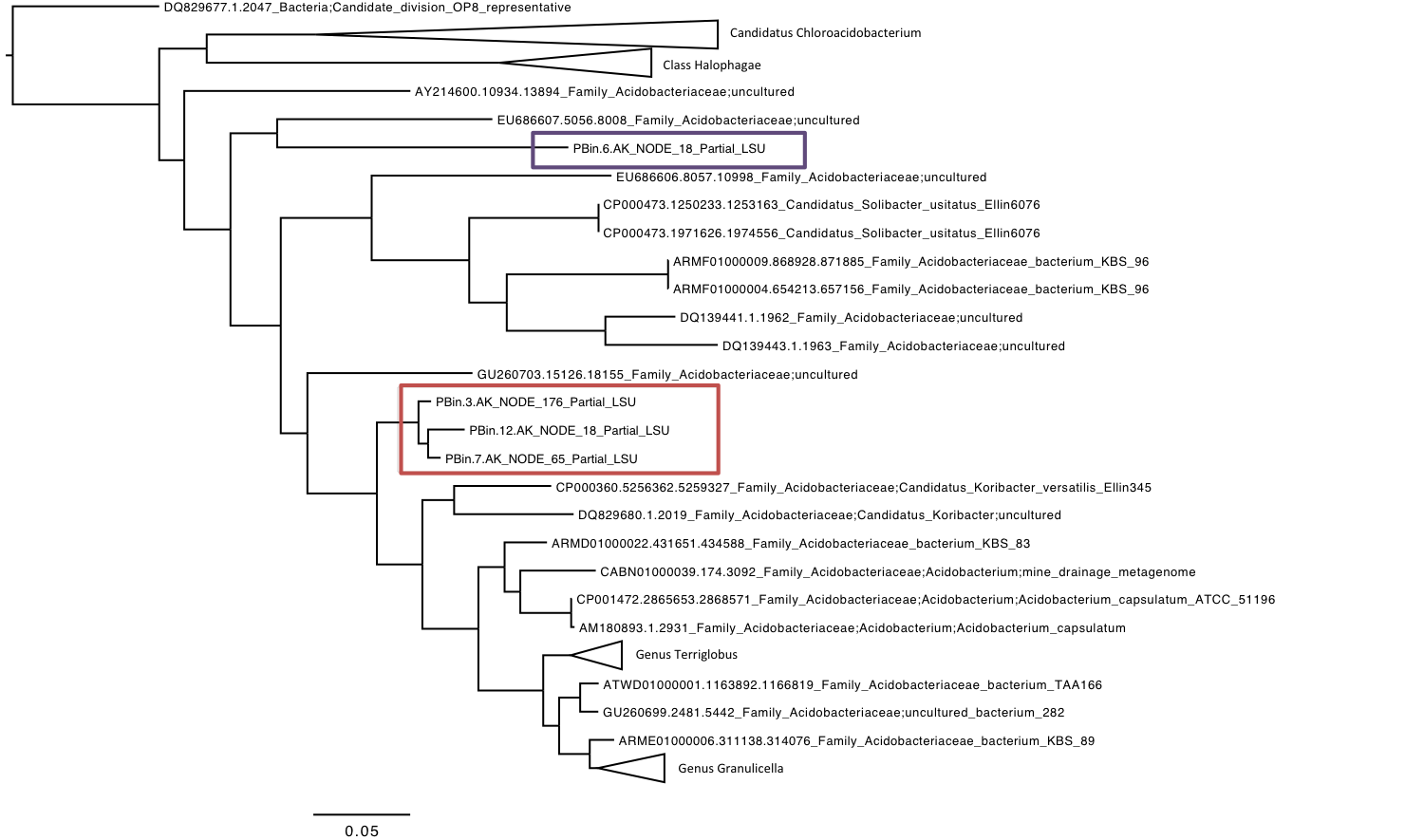


**Supplementary Figure 6 | Phylogenetic tree displaying relatedness of bin assemblies 03, 06, 07, and 11/12 to other known *Acidobacteria* representatives.** Underlying data are aligned LSU sequences from bins and acidobacterial LSU sequences from SILVA LSU database (Quast et al. 2013) using a representative from *Ca. OP8* as an out-group. For many sequences, the SILVA name was modified for simplicity/aesthetics and unique accession numbers are provided in all instances that link many nodes to respective database sequences. Monophyletic groups representing bins are outlined in red (bins 03, 07, 11/12*)* and purple (bin 06).


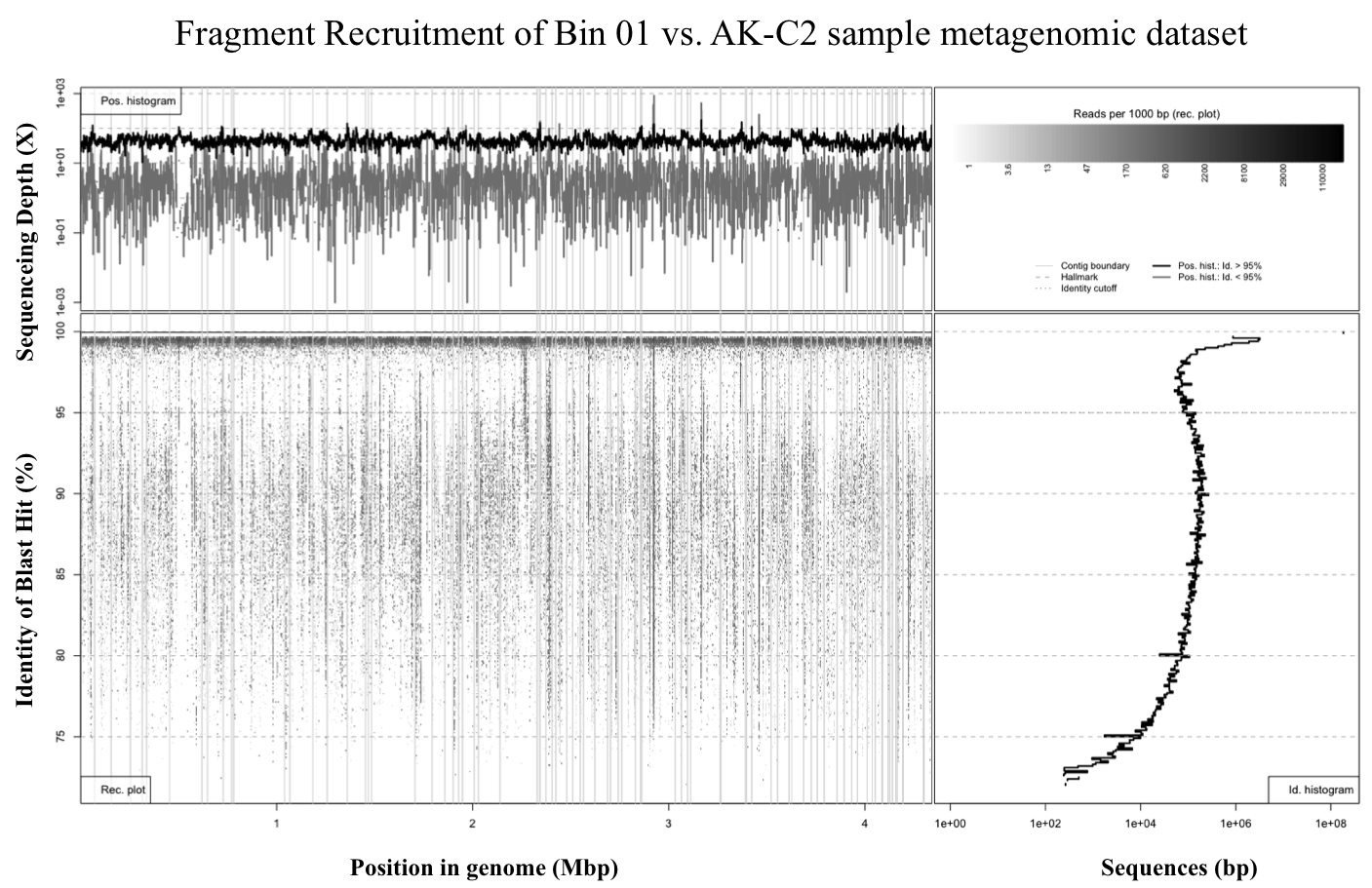

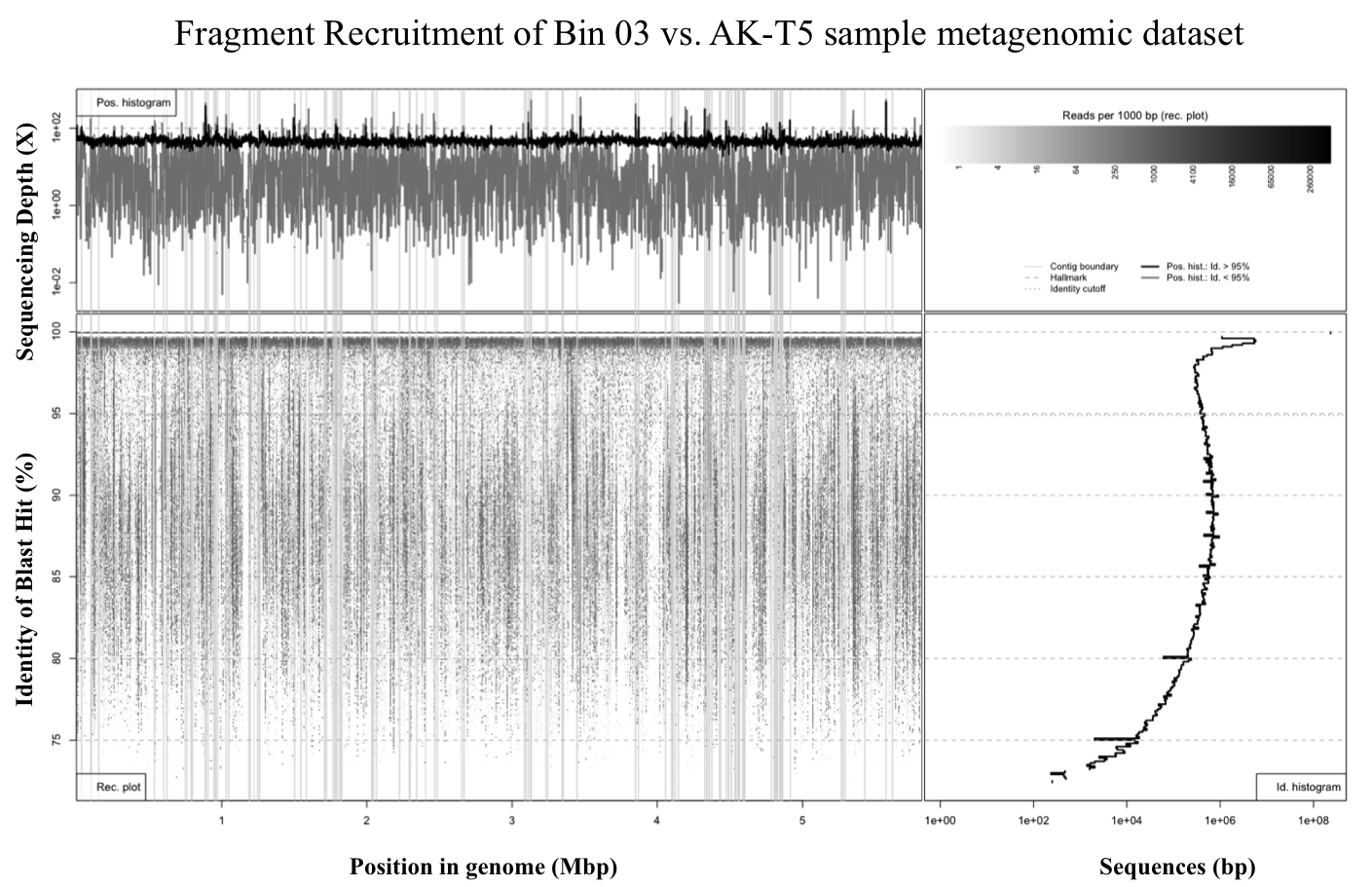


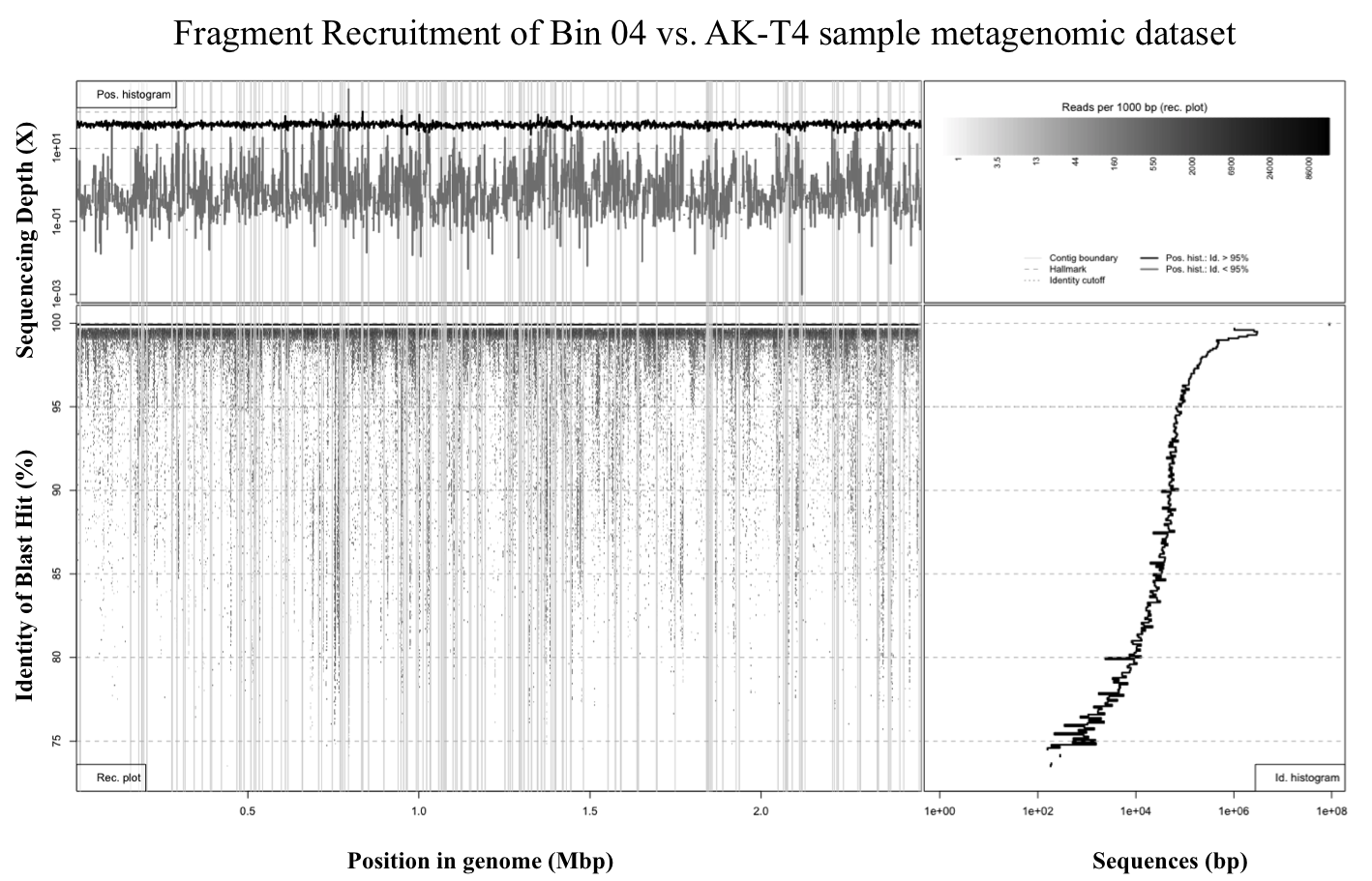


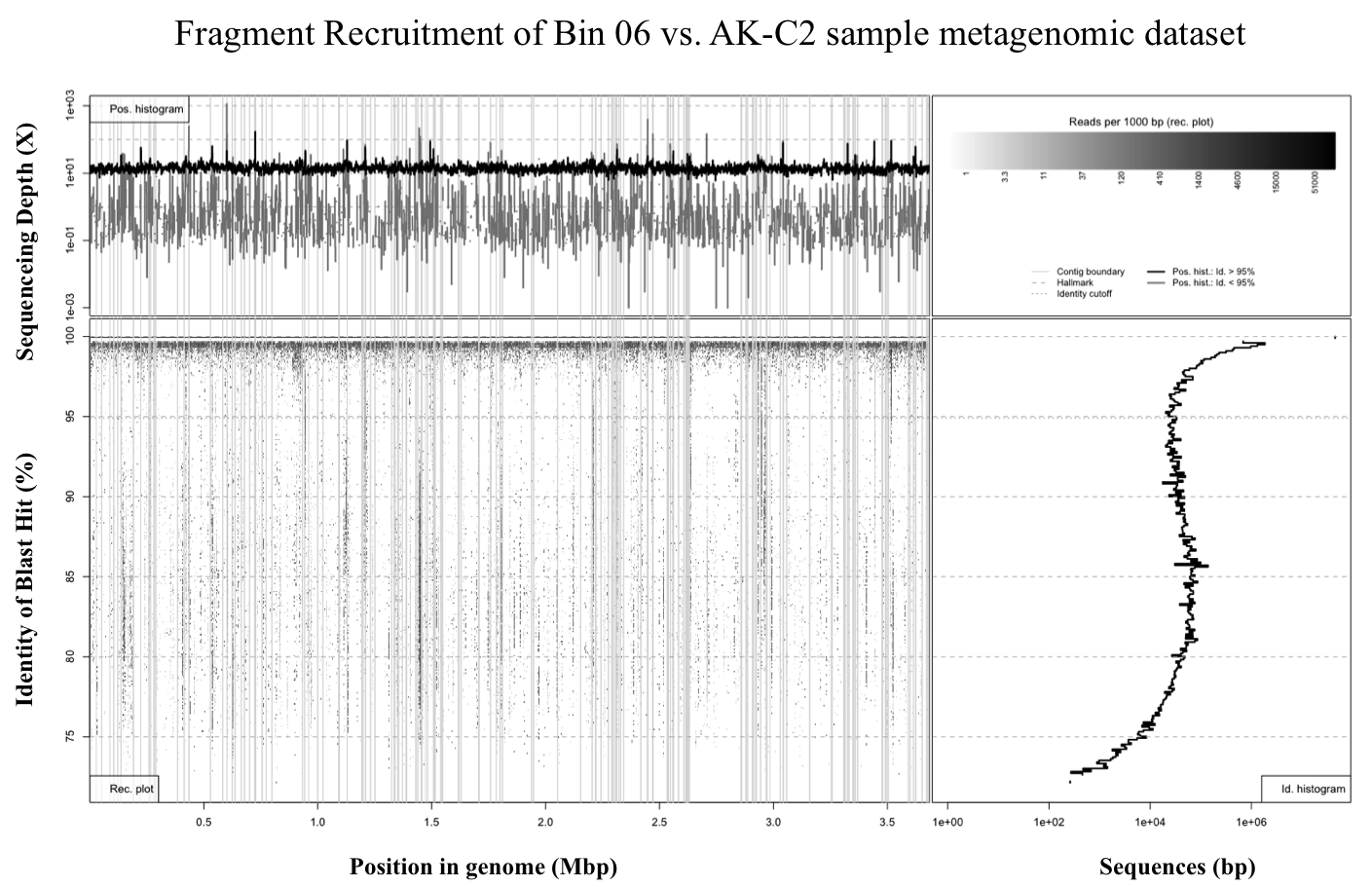


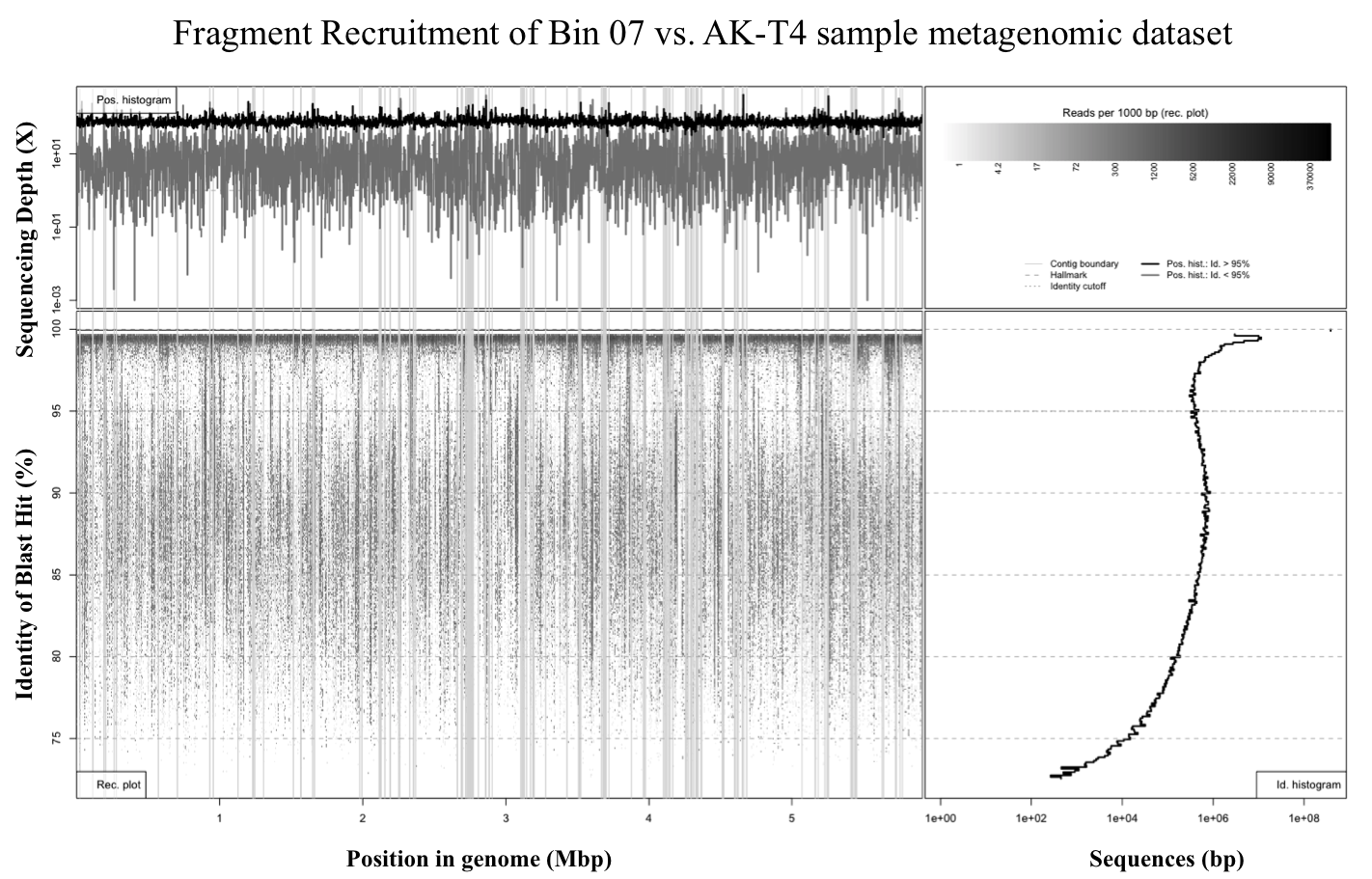


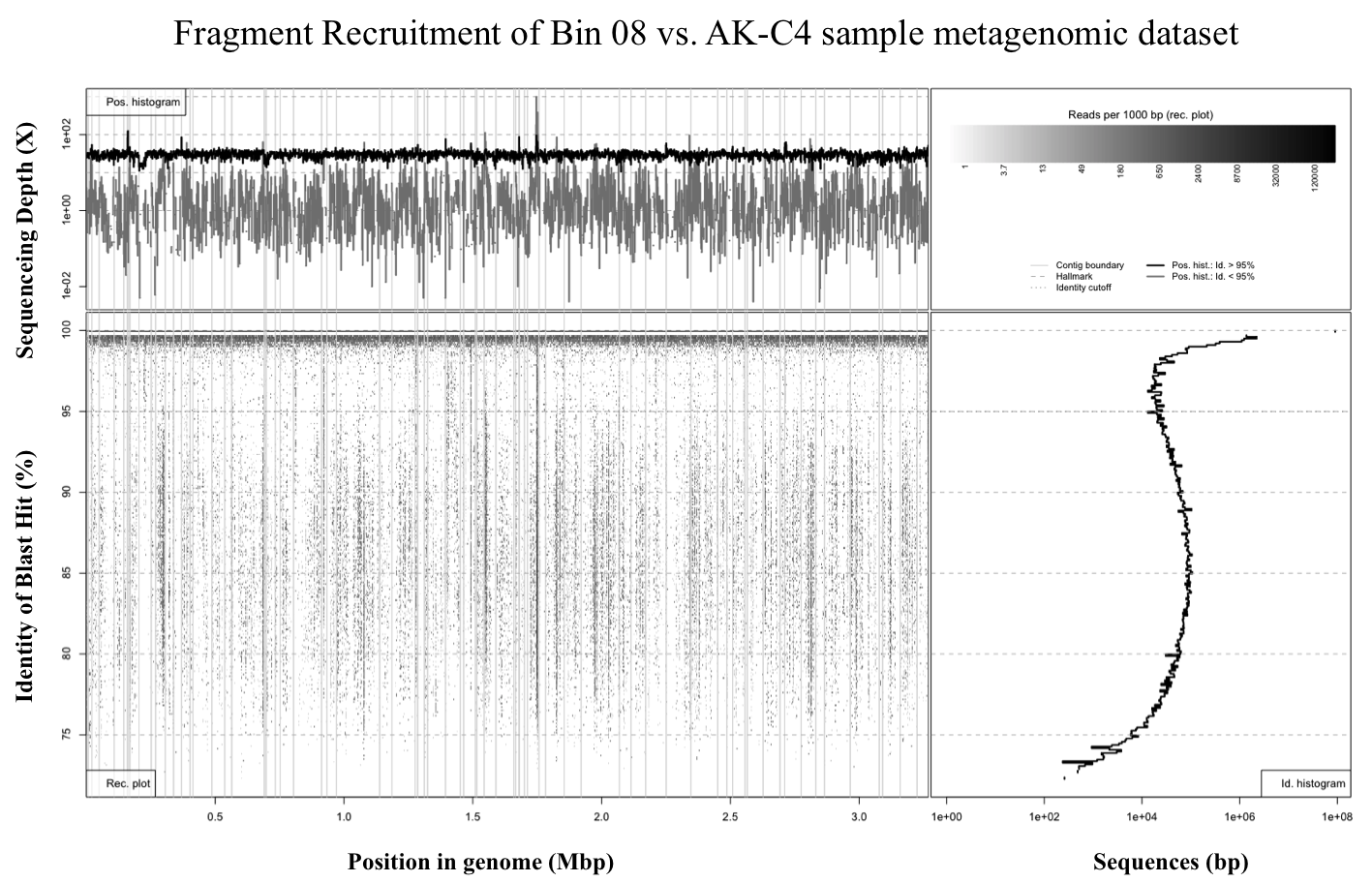


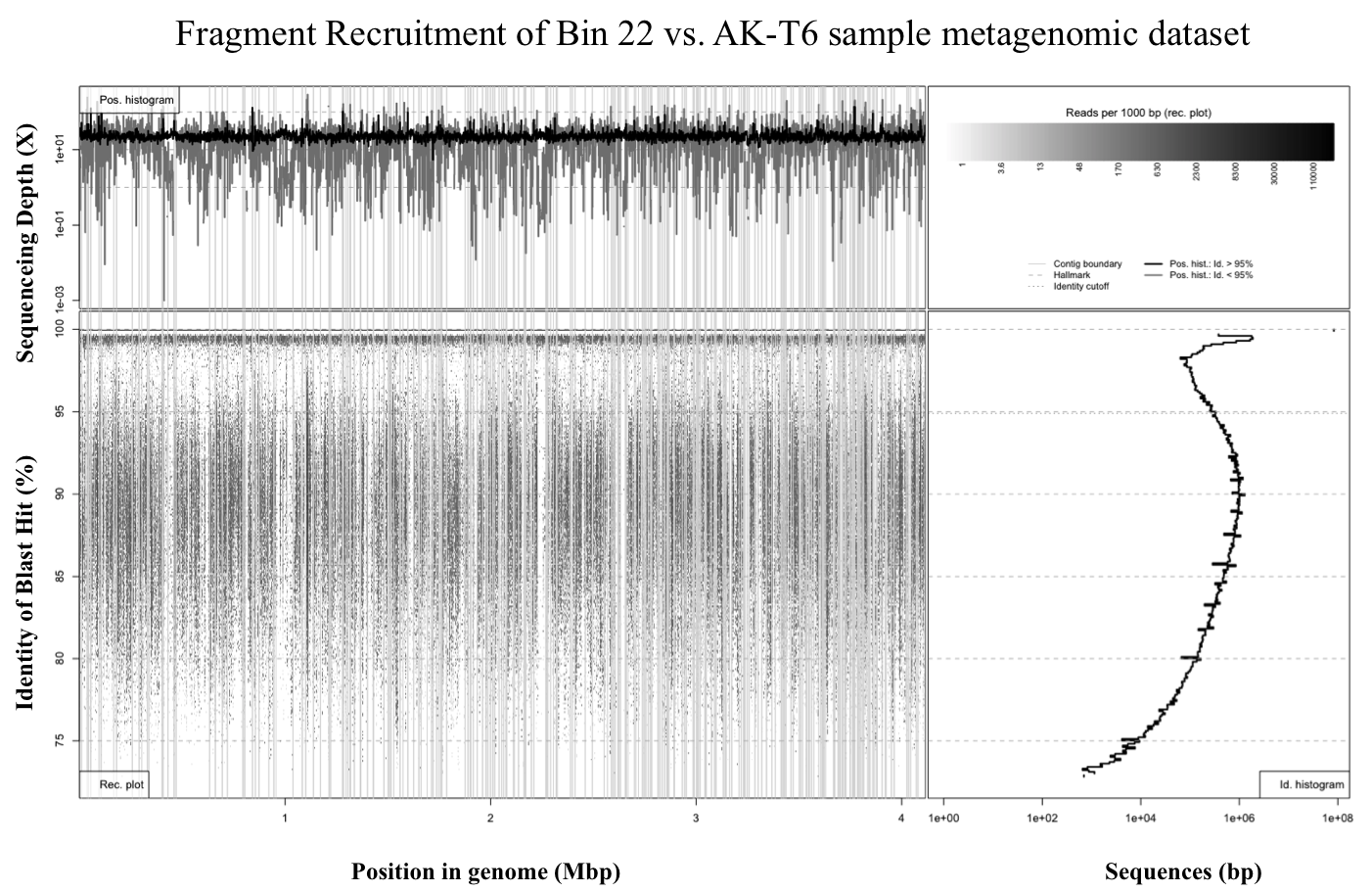


**Supplementary Figure 7 | Fragment recruitment plots.** Diagrams expressing coverage of all contigs of several population bins assembled from AK metagenomes. All metagenomic short reads from select sample metagenomes (usually the sample in which the organism had the highest representation) were searched against each bin in a megablast search. The position histogram (top left of each) displays the coverage of each base position, determined by a 1000bp window average. An even coverage across the entire contig from matching reads that are high identity (>95%) as well as an absence of redundant positions is indicative of a high quality contig from a single population. The recruitment plot (bottom left of each) shows where individual metagenomic reads matched to the population bin and the identity (%) of the match. The ID histogram (bottom right) displays the total number of short read-derived base-positions at given percent identities. Note that in all cases shown, a sequence-discrete population represented by reads showing high nucleotide identity to the reference population genome sequence (typically >95% nucleotide identity) and even coverage across the length of the reference sequence are obvious. In some cases such as Bin 22 against AK-T6 closely related (i.e., showing 80-95% nucleotide identity to the reference sequence) population(s) are also present (co-occurring) in the sample while in cases such as Bin 04 against AK-T4, no co-occurring relatives are present (or those are of very low abundance to be robustly detected by metagenomics)

.


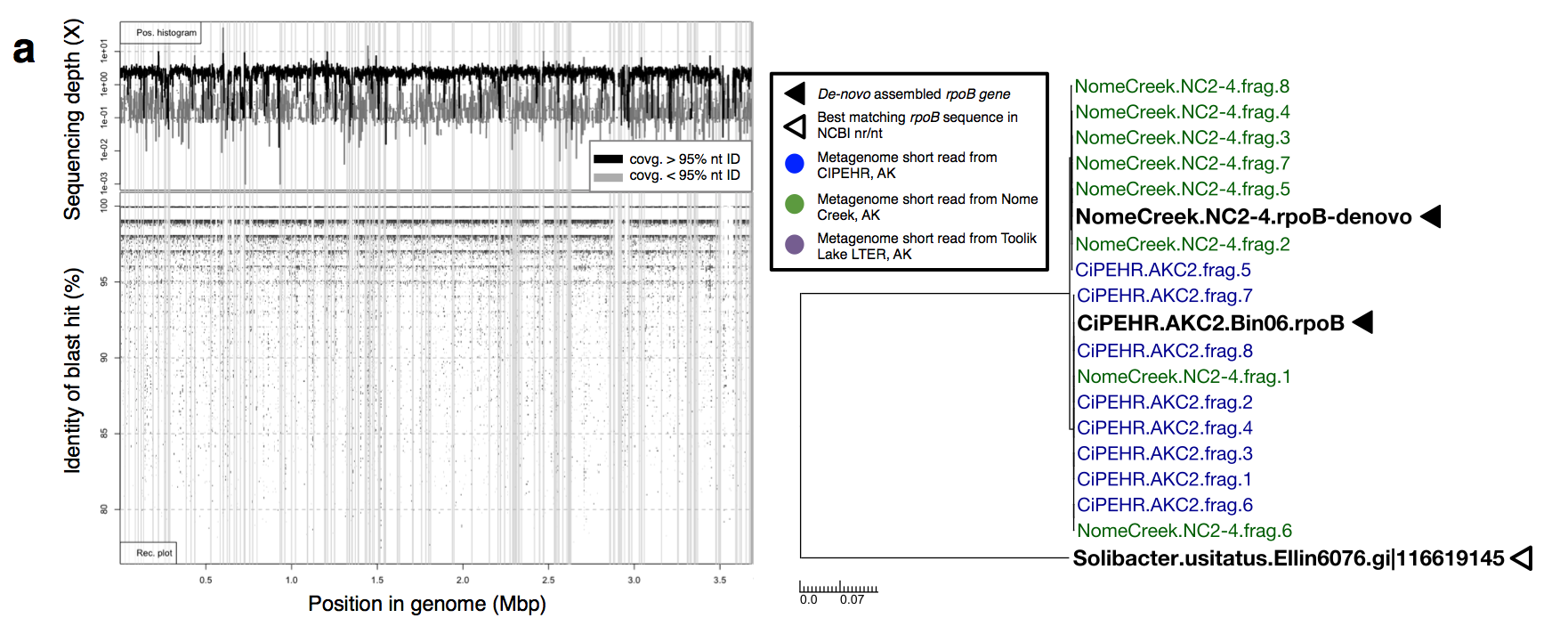


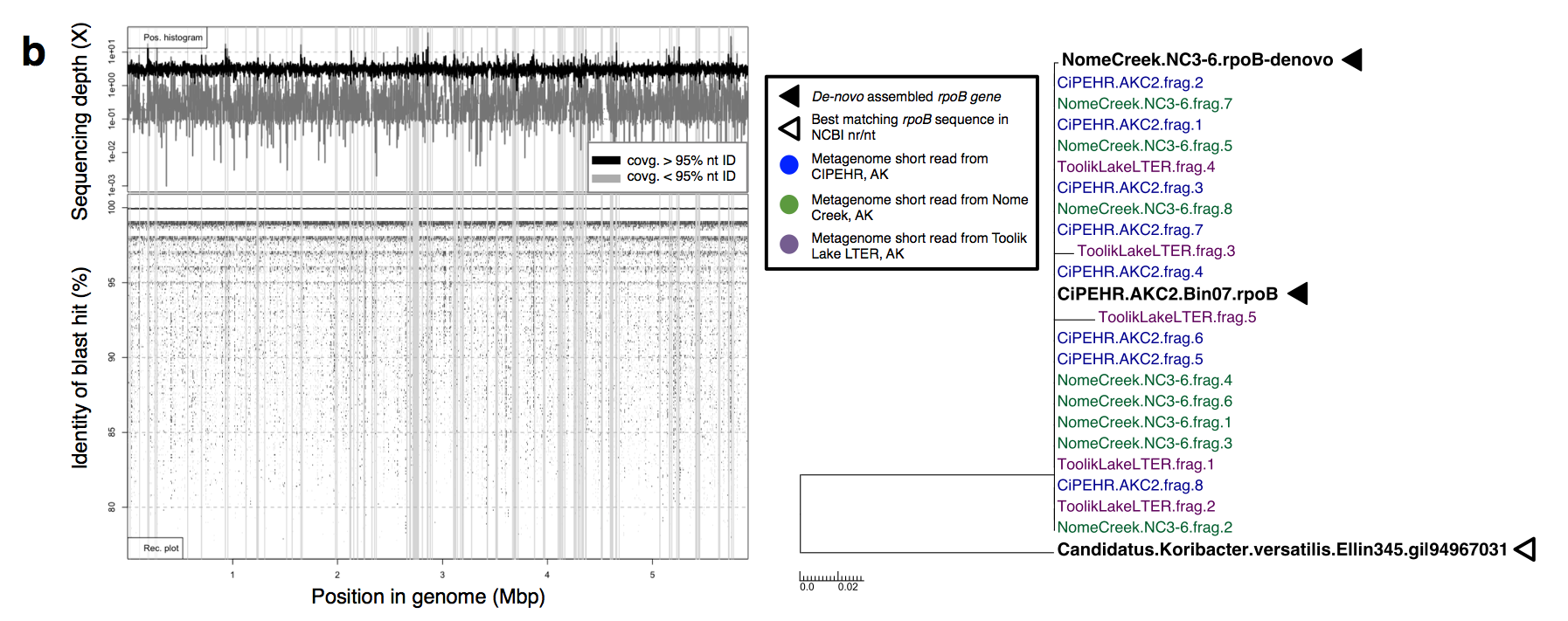

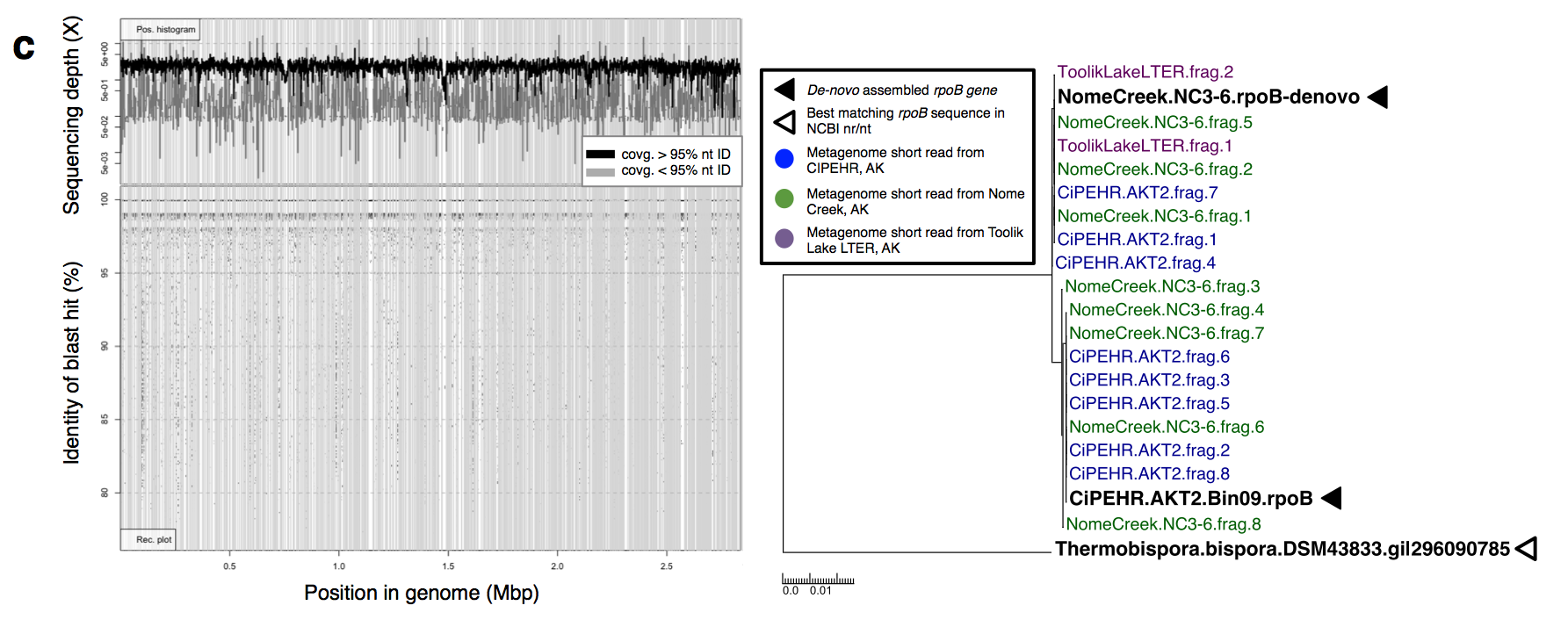


**
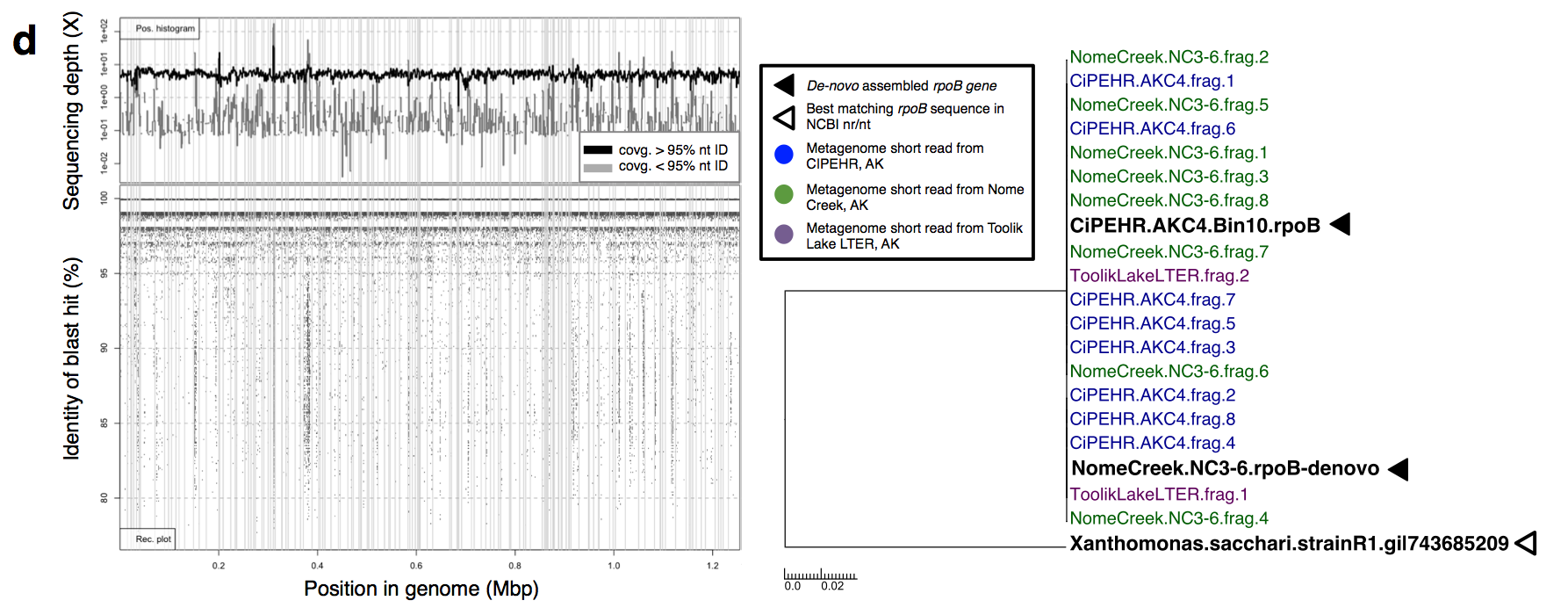
**

**Supplementary Figure 8 | Fragment recruitment plots and phylogenetic trees of *rpoB* gene sequences of four geographically dispersed population bins.** Population bins assembled from CiPEHR metagenomic datasets were searched against publically available metagenomes representing distant locations for fragment recruitment analysis in the same manner as discussed in Figure S7. Diagrams express the coverage of concatenated contigs (from left to right; contigs separated by grey vertical bars) in metagenomes from Taş et al. 2014 (Nome Creek, AK; >200km distance from CiPEHR). Phylogenetic trees constructed from *rpoB* gene sequences assembled, *de-novo,* from metagenomes at both locations independently (in boldface) as well as unassembled *rpoB*-encoding short reads from the same metagenome. Trees also include a reference *rpoB* gene sequence from NCBI (outgroup) as well as unassembled reads from a soil metagenome from Fierer et al. 2012 (Toolik Lake LTER, AK; ~530km distance from CiPEHR). The trees reveal the high relatedness among organisms present at all three sites (e.g., reads do not cluster by site but are intermixed). Diagrams represent (**a**) Sample NC2-4 (Burned 10-20cm) vs Bin 06 assembly, (**b**) Sample NC3-6 (Control 50-60cm) vs Bin 07 assembly, (**c**) Sample NC3-6 vs Bin 09 assembly, (**d**) and Sample NC3-6 vs Bin 10 assembly.


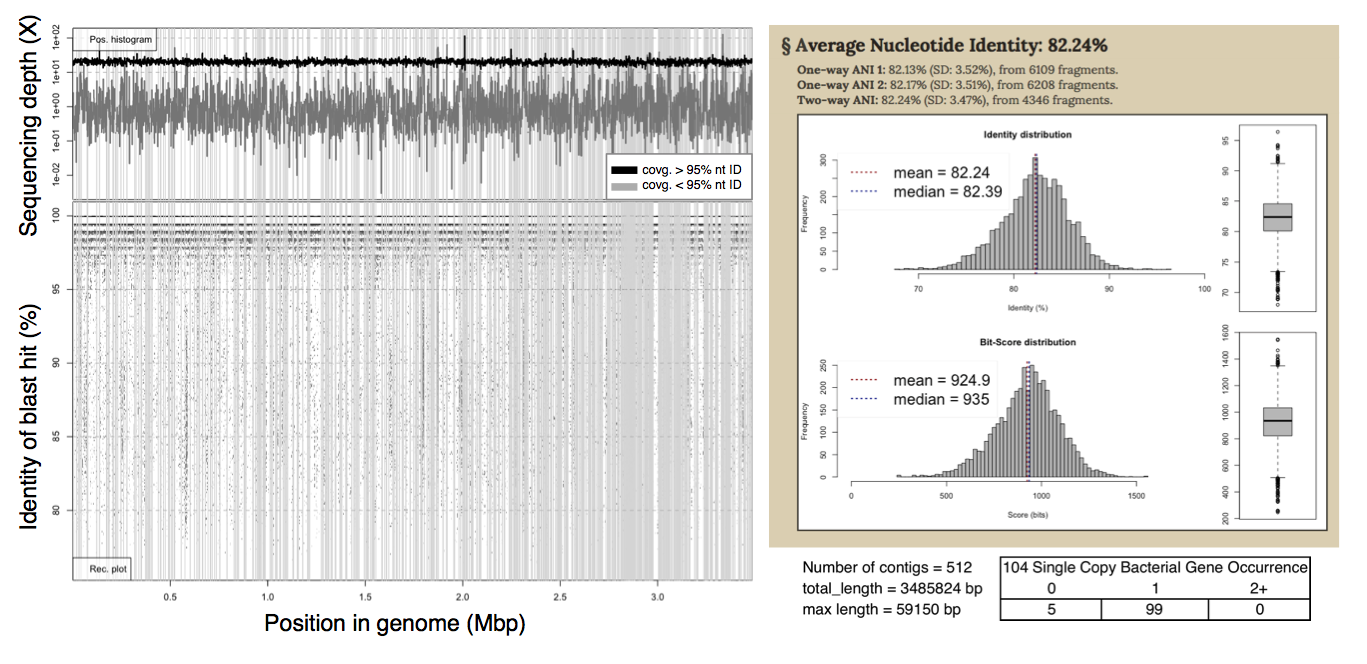


**Supplementary Figure 9 | The acidobacterial population recovered from the SPRUCE metagenome.** Graphs show the acidobacterial fragment recruitment plot of SPRUCE soil metagenome assembly (left) average nucleotide identity comparison to bin 11 (right top), basic assembly statistics, and assessment of bin completeness and contamination (bottom right table). Population bin assembled from SPRUCE soil metagenomes were searched (with megablast) against the dataset from which it was derived (NCBI SRA entry SRR1157608; 75-100 cm soil depth) and a fragment recruitment plot was generated in the same manner as discussed in Figure S5. Average nucleotide identity (ANI) comparison was made between this assembled bin and the closest matching assembly from CiPEHR metagenomes, bin 11, using web-based ANI calculation tools available at http://enve-omics.ce.gatech.edu/ani/. Presence of 104 single copy bacterial genes, which is used in the assessment of assembly completion and contamination, was performed using CheckM.


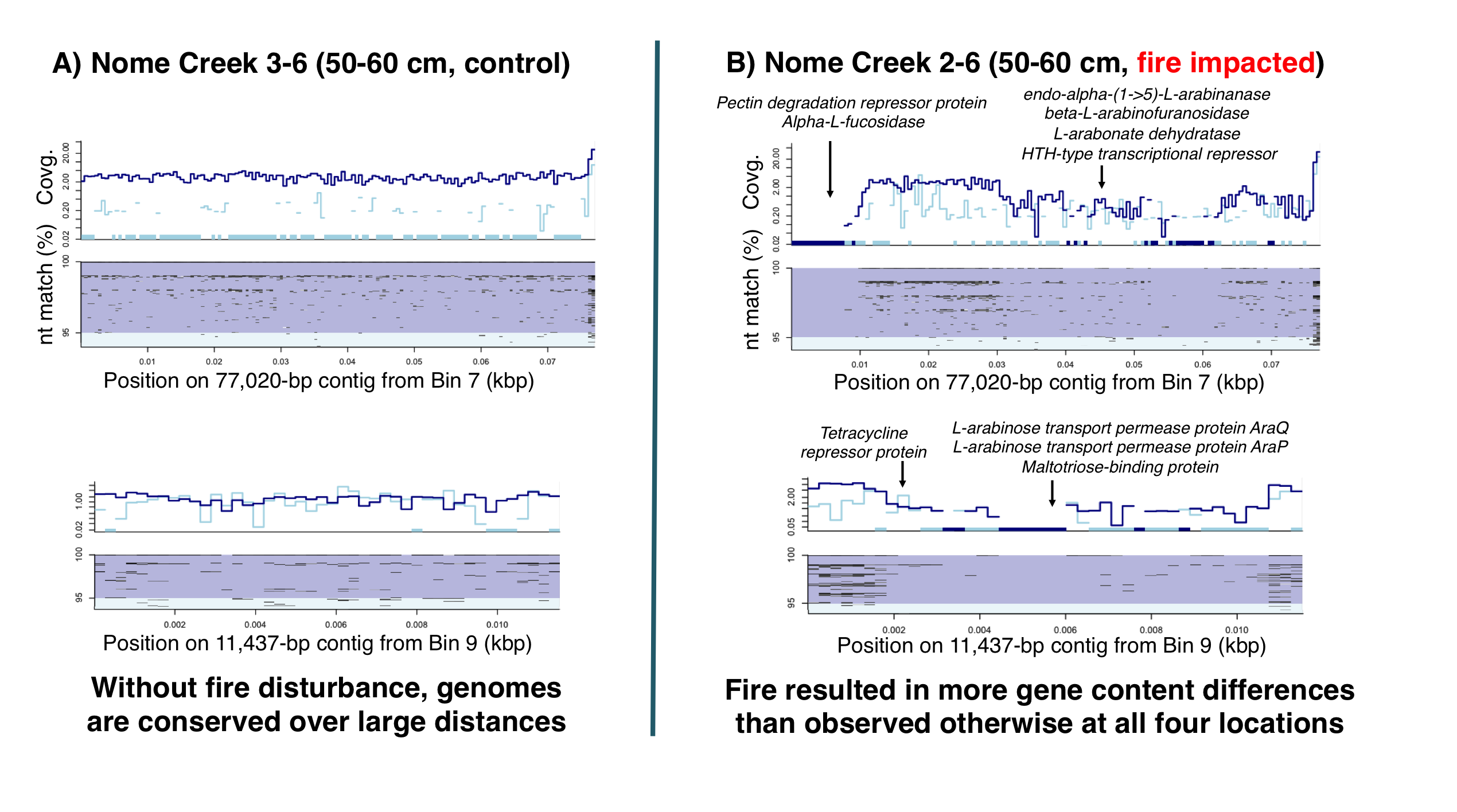


**Supplementary Figure 10 | Contigs from two populations conserved between CiPEHR and Nome Creek control soils, but with gene loss resulting from fire event at Nome Creek.** Contigs from population bins assembled from CiPEHR metagenomic datasets were searched against publically available metagenomes representing A) Nome Creek control 50-60cm depth and B) Nome Creek fire impacted 50-60cm depth) for fragment recruitment analysis in the same manner as discussed in Figure S7. Gene content difference was 2-3% of the entire genome assembly in the case of bins 7 and 9. Contigs shown above displayed higher than usual gene loss and thus, are displayed for contrast.

**Supplementary Table 1 | Soil chemical and physical measurements for CiPEHR and KFFL soil samples.** Method descriptions for each type of measurement can be found in Xue et al. 2016.


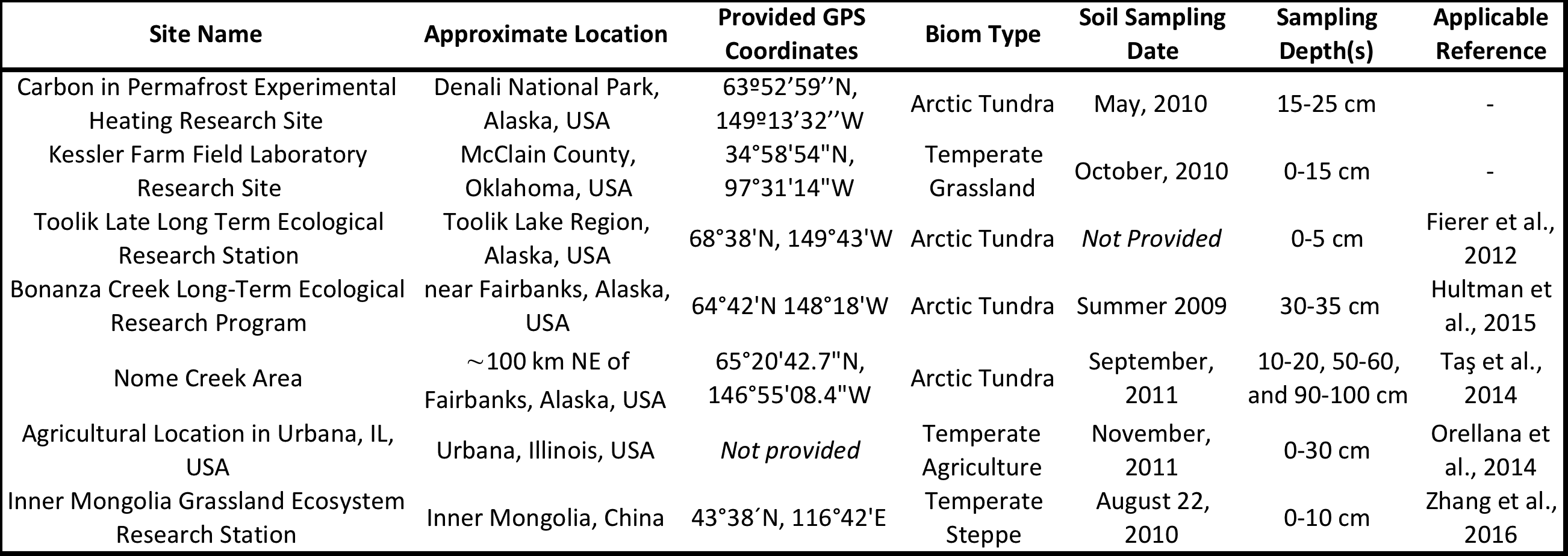


**Supplementary Table 2 | Datasets (including pertinent information) used to compare community characteristics (taxa, populations, functional genes) shared within Alaskan tundra soils.**


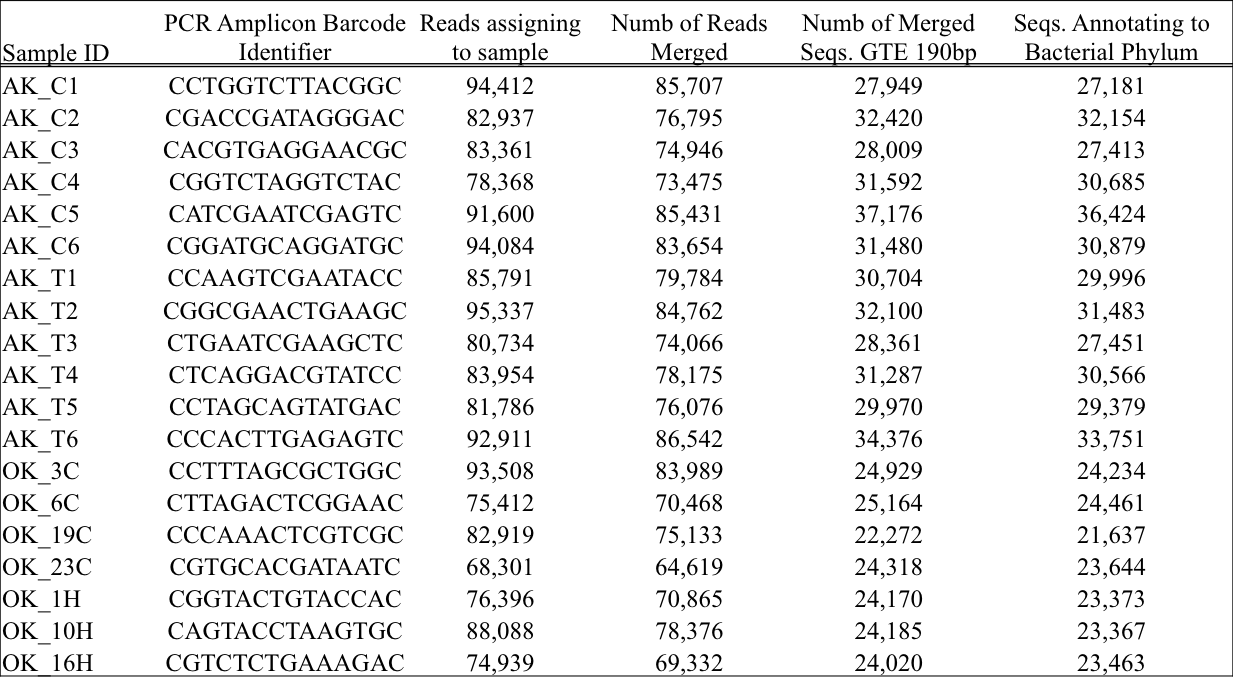


**Supplementary Table 3 | Information pertaining to sample-specific barcode identifiers, the merge-ability, sequence quality, and number of annotation made for 16S rRNA gene amplicon sequences representative of each soil sample.** For sample ‘OK_20H’, PCR amplicon data were not available. Therefore, it was excluded from this portion of the analyses.

| Sample ID | Total Number of Raw Seqs. | Total Sequencing depth (bp) | Number of Merged Seqs. | Merged Seqs. GTE 100bp After Trimming | Seqs. Annotated to a SwissProtKB Gene | Perc. of Query Seqs. Annotated |
| --- | --- | --- | --- | --- | --- | --- |
| AK_C2 | 140,384,743 | 42,115,422,900 | 88,938,535 | 81,911,488 | 11,272,986 | 13.76% |
| AK_C3 | 127,668,458 | 38,300,537,400 | 55,494,410 | 50,258,776 | 7,133,996 | 14.19% |
| AK_C4 | 123,994,800 | 37,198,440,000 | 43,931,094 | 41,215,414 | 7,007,438 | 17.00% |
| AK_C5 | 136,918,270 | 41,075,481,000 | 77,972,852 | 74,233,948 | 11,093,641 | 14.94% |
| AK_C6 | 145,020,548 | 43,506,164,400 | 88,303,693 | 79,740,381 | 9,816,350 | 12.31% |
| AK_T1 | 88,621,343 | 26,586,402,900 | 43,063,960 | 41,082,354 | 6,082,884 | 14.81% |
| AK_T2 | 115,149,822 | 34,544,946,600 | 52,812,203 | 47,829,930 | 6,689,572 | 13.99% |
| AK_T3 | 129,728,436 | 38,918,530,800 | 70,847,511 | 65,415,616 | 9,846,729 | 15.05% |
| AK_T4 | 144,973,423 | 43,492,026,900 | 110,018,815 | 101,786,723 | 12,711,784 | 12.49% |
| AK_T5 | 166,411,844 | 49,923,553,200 | 110,104,603 | 100,970,305 | 12,602,459 | 12.48% |
| AK_T6 | 149,370,367 | 44,811,110,100 | 109,824,451 | 99,923,813 | 12,167,769 | 12.18% |
| OK_3C | 120,253,701 | 36,076,110,300 | 52,477,090 | 48,578,565 | 8,036,148 | 16.54% |
| OK_6C | 109,642,544 | 32,892,763,200 | 64,581,913 | 20,384,797 | 3,266,908 | 16.03% |
| OK_19C | 132,777,103 | 39,833,130,900 | 65,725,755 | 60,307,185 | 9,293,533 | 15.41% |
| OK_23C | 116,865,558 | 35,059,667,400 | 57,197,196 | 52,759,317 | 8,589,159 | 16.28% |
| OK_1H | 146,224,604 | 43,867,381,200 | 70,075,328 | 64,873,555 | 10,775,911 | 16.61% |
| OK_10H | 135,467,362 | 40,640,208,600 | 63,057,030 | 58,630,367 | 9,609,617 | 16.39% |
| OK_16H | 141,761,229 | 42,528,368,700 | 38,561,786 | 31,704,520 | 4,452,998 | 14.05% |
| OK_20H | 123,179,662 | 36,953,898,600 | 71,373,159 | 65,363,171 | 10,388,444 | 15.89% |

**Supplementary Table 4 | Information pertaining to the merge-ability, sequence quality, and number/percent of annotation made for shotgun-metagenome datasets representing each soil sample.**

**Supplementary Table 5 | Soil microbial community complexity for estimations as determined by Nonpareil.** Nonpareil is a statistical tool that uses read redundancy to estimate dataset complexity and the amount of sequencing effort needed to achieve a desired level of åcoverage, given for each sample, as well as ‘comprehensive datasets’ containing all reads from either site.


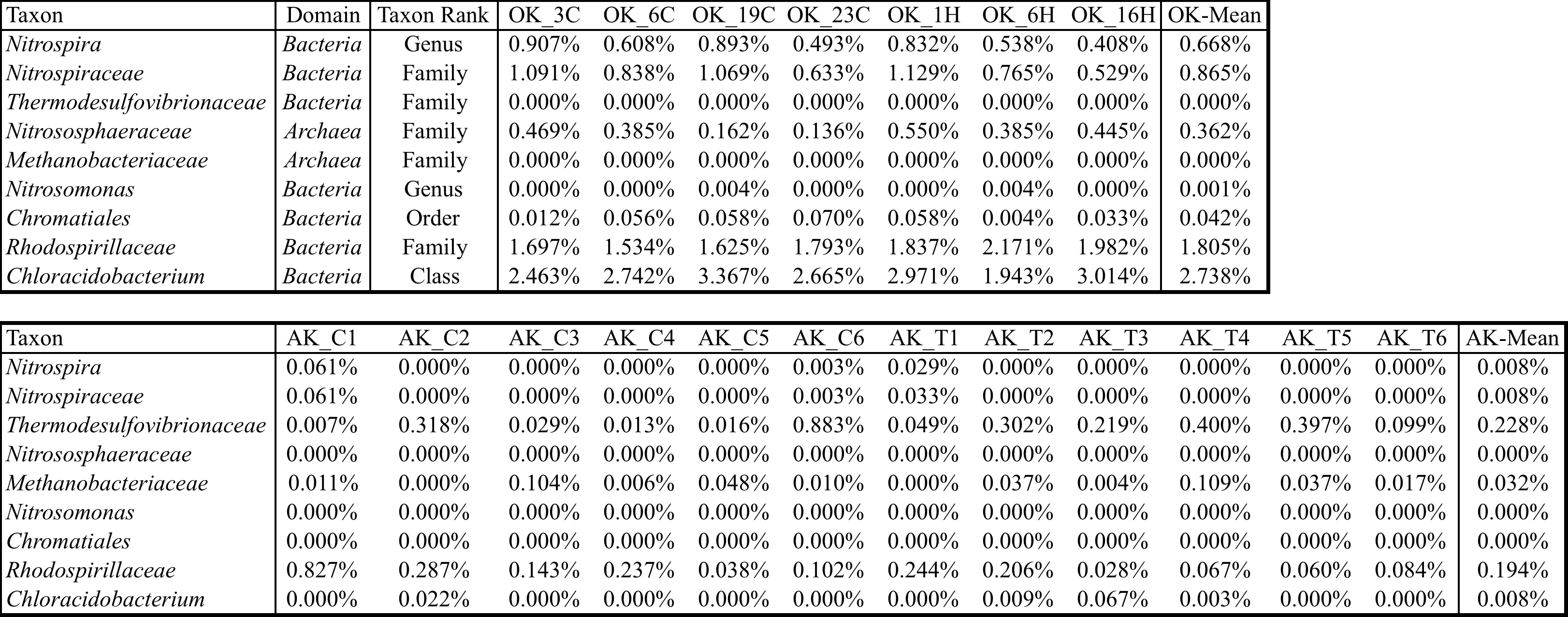


**Supplementary Table 6 | The Abundance of *Bacteria* and *Archaea* taxonomic groups of interest with means specific to each study site.** Data shown in table was determined by 16S rRNA gene amplicons of the V4 region. Oklahoma sample 20H is omitted from this analysis due to technical difficulties during library preparation.


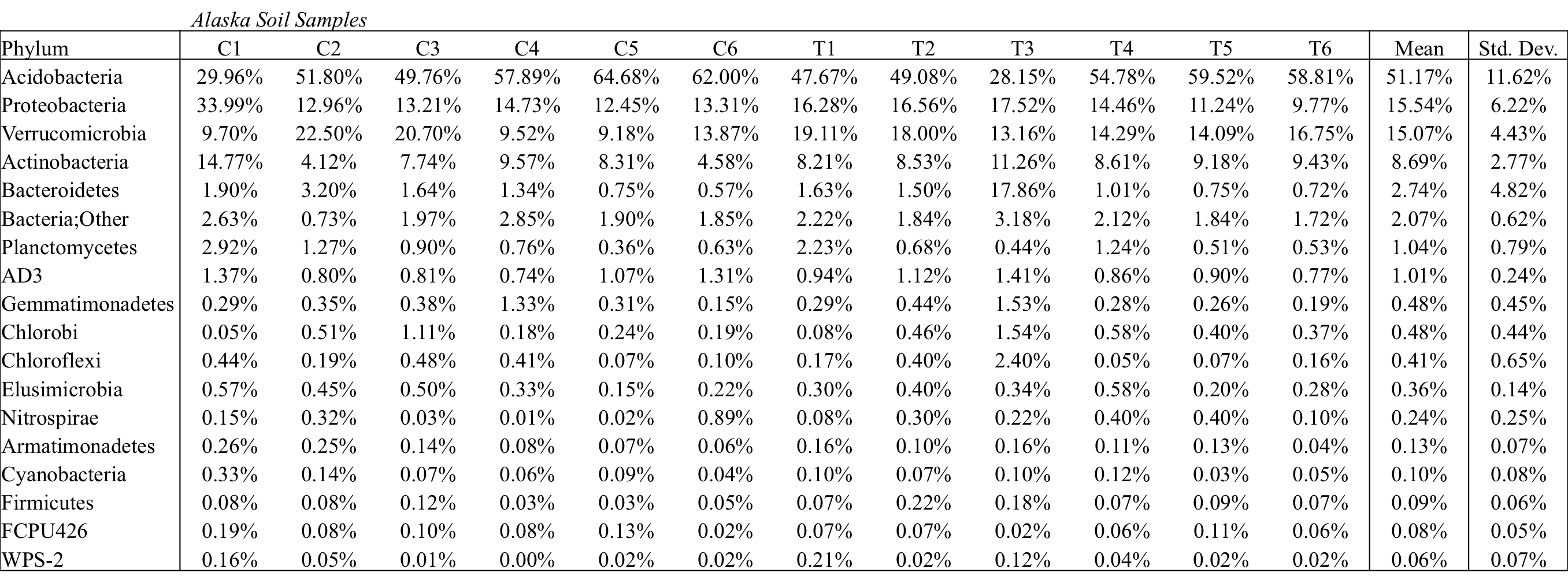

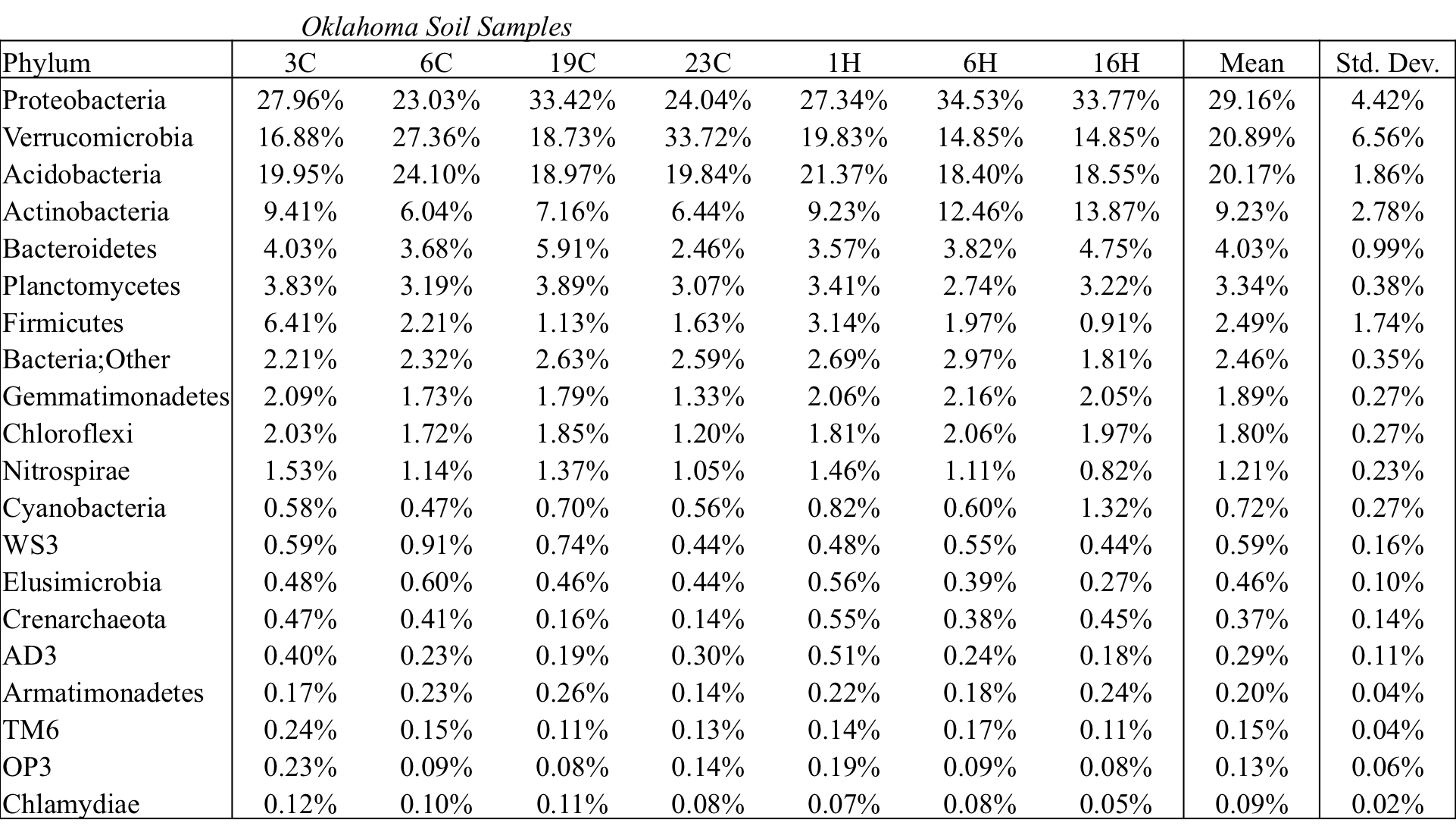


**b**

**a**

**Supplementary Table 7 | Abundance of Bacterial and Archaeal phyla in (a) Alaskan tundra CiPHER site soils, and (b) Oklahoma prairie KFFL site soils.** Means and standard deviations specific to each site are displayed. Data shown in table was determined by 16S rDNA PCR amplicons of the V4 region. Phyla are in order from highest to lowest mean for each site. Phyla with a mean abundance of less than 0.5% are omitted from each table. Note that Oklahoma sample 20H is omitted from this analysis due to technical difficulties during library preparation.


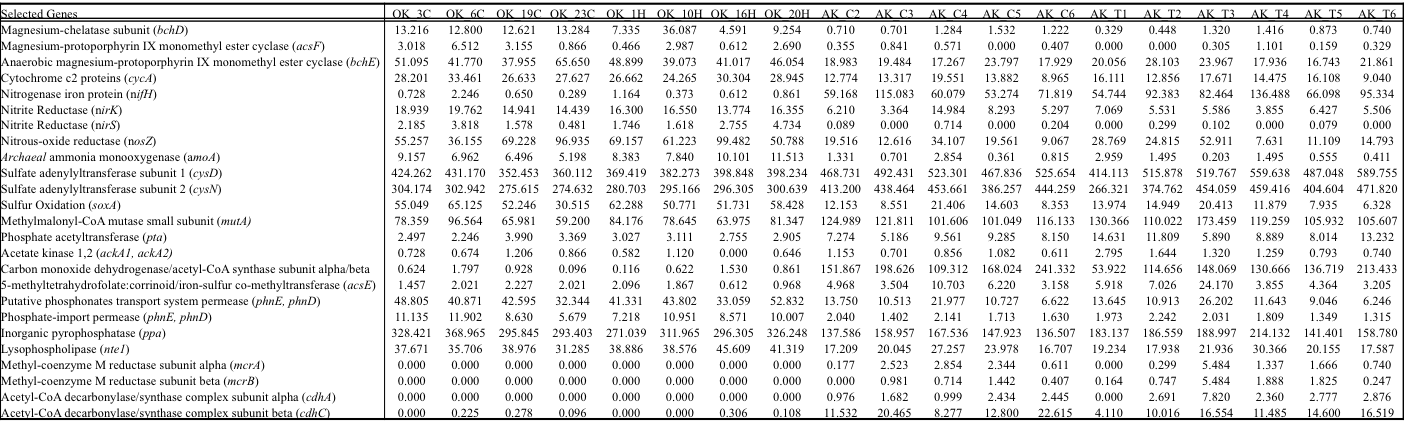


**Supplementary Table 8 | The relative abundance of selected genes in each metagenomic dataset that were specifically mentioned in the text.** Values represent counts out of 1,000,000 annotations made. Gene descriptions and IDs correspond to those given in the Swiss-Prot protein database (The Uniprot Consortium).

|  | POPULATION BIN BASIC INFO | | | | 104 SINGLE COPY BACTERIAL GENES | | | |  |  |
| --- | --- | --- | --- | --- | --- | --- | --- | --- | --- | --- |
| Bin ID | n50 (bp) | Longest (bp) | Total Length (bp) | Number Contigs | 0 | 1 | 2 | 3+ | Estimated Completeness | Estimated Contamination |
| 1 | 87585 | 258416 | 4341484 | 85 | 5 | 98 | 1 | 0 | 94.0% | 1.7% |
| 2 | 44196 | 144885 | 4793253 | 228 | 21 | 83 | 0 | 0 | 89.7% | 0.0% |
| 3 | 167654 | 415574 | 5822639 | 110 | 4 | 100 | 0 | 0 | 95.7% | 0.0% |
| 4 | 36041 | 146424 | 2468837 | 156 | 14 | 90 | 0 | 0 | 81.0% | 0.0% |
| 5 | 20203 | 76412 | 2053920 | 189 | 12 | 92 | 0 | 0 | 83.5% | 0.0% |
| 6 | 54187 | 226195 | 3684989 | 154 | 2 | 102 | 0 | 0 | 97.4% | 0.0% |
| 7 | 142525 | 388009 | 5910590 | 149 | 12 | 92 | 0 | 0 | 89.8% | 0.0% |
| 8 | 65109 | 167976 | 3268372 | 73 | 3 | 100 | 1 | 0 | 95.7% | 1.7% |
| 9 | 5369 | 29935 | 3892756 | 976 | 69 | 34 | 1 | 0 | 54.3% | 1.7% |
| 10 | 12295 | 50046 | 2301754 | 289 | 18 | 85 | 1 | 0 | 81.3% | 1.7% |
| 11 | 51987 | 197629 | 5172412 | 191 | 2 | 102 | 0 | 0 | 98.8% | 0.0% |
| 12 | 58175 | 161485 | 2468333 | 73 | 80 | 24 | 0 | 0 | 37.1% | 0.0% |
| 13 | 10550 | 36899 | 3730785 | 564 | 27 | 75 | 2 | 0 | 66.6% | 3.5% |
| 14 | 11076 | 60265 | 4040674 | 575 | 39 | 64 | 1 | 0 | 65.7% | 1.7% |
| 15 | 4362 | 19644 | 2402663 | 675 | 58 | 46 | 0 | 0 | 53.0% | 0.0% |
| 16 | 16102 | 93823 | 5151448 | 478 | 57 | 46 | 1 | 0 | 68.7% | 1.7% |
| 17 | 6223 | 29378 | 3210355 | 712 | 52 | 52 | 0 | 0 | 48.3% | 0.0% |
| 18 | 12136 | 64006 | 7779547 | 979 | 44 | 52 | 8 | 0 | 72.1% | 13.8% |
| 19 | 9878 | 99463 | 4721202 | 820 | 45 | 58 | 1 | 0 | 65.5% | 1.7% |
| 20 | 13258 | 53656 | 3231835 | 375 | 31 | 71 | 2 | 0 | 83.1% | 1.0% |
| 21 | 6777 | 61204 | 3385891 | 718 | 41 | 62 | 1 | 0 | 55.4% | 1.7% |
| 22 | 27045 | 163169 | 4115219 | 235 | 30 | 74 | 0 | 0 | 74.4% | 0.0% |
| 23 | 20762 | 49785 | 1603225 | 119 | 49 | 55 | 0 | 0 | 44.1% | 0.0% |
| 24 | 218163 | 218163 | 388312 | 3 | 104 | 0 | 0 | 0 | 0.0% | 0.0% |
| 25 | 13861 | 63010 | 585735 | 55 | 103 | 1 | 0 | 0 | 0.9% | 0.0% |
| 26 | 15154 | 70312 | 961897 | 108 | 102 | 2 | 0 | 0 | 3.5% | 0.0% |
| 27 | 26244 | 147429 | 8218361 | 566 | 3 | 101 | 0 | 0 | 94.8% | 0.0% |

**Supplementary Table 9 | Summary statistics for each bin.** Including n50 (*i.e.*, the length that >50% of the assembly is in contigs of this length or longer), the length of the longest contig, the total length of all contigs combined, and the number of contigs comprising a bin. All contigs were > 1kbp. CheckM was used to assess each bin for completedness, checking for the presence of 104 single copy bacterial genes. The number in each column represents the number of instances each of the 104 Bacterial single copy genes was found in each bin (i.e. 98 single copy genes were found once in Bin 01). Bins highlighted in green indicate those estimated to be >80% complete. Single copy marker genes that occur more than once serve as an indicator of contamination – *i.e.* that the assembly is combined with sequences from a more than one organism.

| Bin ID | Length (bp) | ID Match (%) | Best Match (SILVA LSU database v119) | Database accession ID |
| --- | --- | --- | --- | --- |
| 1 | 2872 | 93.04 | Verrucomicrobia;Opitutae;Opitutales;Opitutaceae;Opitutus;Opitutus terrae PB90-1 | CP001032.2053384.2056251 |
| 3 | 568 | 94.37 | Acidobacteria;Acidobacteriales;Acidobacteriaceae;Acidobacteriaceae bacterium KBS 83 | ARMD01000022.431651.434588 |
| 6 | 1546 | 80.21 | Acidobacteria;Acidobacteriales;Acidobacteriaceae;Candidatus Koribacter;Candidatus Koribacter versatilis Ellin345 | CP000360.5256362.5259327 |
| 7 | 358 | 94.69 | Acidobacteria;Acidobacteriales;Acidobacteriaceae;Granulicella;Granulicella mallensis MP5ACTX8 | CP003130.693578.696525 |
| 8 | 3001 | 93.37 | Actinobacteria;Thermoleophilia;Solirubrobacterales;Conexibacteraceae;Conexibacter;Conexibacter woesei DSM 14684 | CP001854.3761310.3764306 |
| 10 | 996 | 84.74 | Proteobacteria;Gammaproteobacteria;Legionellales;Coxiellaceae;Coxiella;Pseudomonas moraviensis R28-S | AYMZ01000007.155400.157795 |
| 12 | 487 | 94.25 | Acidobacteria;Acidobacteria;Acidobacteriales;Acidobacteriaceae;Acidobacteriaceae bacterium KBS 83 | ARMD01000022.431651.434588 |
| 27 | 3022 | 88.05 | Bacteria;Proteobacteria;Deltaproteobacteria;Myxococcales;Polyangiaceae;Sorangium;Sorangium cellulosum So ce56 | AM746676.3488030.3491045 |
|  |  |  |  |  |
| Bin ID | Length (bp) | ID Match (%) | Best Match (SILVA SSU database v119) | Database accession ID |
| 1 | 1484 | 98.99 | Verrucomicrobia;Opitutae;Opitutales;Opitutaceae;Opitutus | JQ311870.1.1484 |
| 2 | 319 | 99.37 | Acidobacteria;Acidobacteriales;Acidobacteriaceae | GQ339162.1.1463 |
| 3 | 338 | 99.41 | Acidobacteria;Subgroup 3;Unknown Family;Candidatus Solibacter | EF018719.1.1346 |
| 4 | 760 | 95.66 | Chlorobi;Ignavibacteria;Ignavibacteriales | FR667819.1.1489 |
| 8 | 1498 | 99.07 | Actinobacteria;Thermoleophilia;Solirubrobacterales;TM146 | EU861899.1.1498 |
| 10 | 973 | 98.77 | Proteobacteria;Gammaproteobacteria;Xanthomonadales | AY963465.1.1458 |
| 14 | 254 | 97.24 | Actinobacteria;Actinobacteria;Frankiales;Acidothermaceae;Acidothermus | AB821057.1.1475 |
| 20 | 169 | 100 | Acidobacteria;Acidobacteriales;Acidobacteriaceae (Subgroup 1) | FJ624922.1.1488 |
| 22 | 241 | 99.59 | Acidobacteria;Acidobacteriales;Acidobacteriaceae (Subgroup 1) | GQ339162.1.1463 |
| 27 | 1557 | 96.34 | Proteobacteria;Deltaproteobacteria;Myxococcales;Polyangiaceae;Sorangium | EU445217.1.1547 |

**Supplementary Table 10 | Information on bins containing ribosomal sequences.** 16S rRNA gene matches were determined by using a BLAST search (word size = 18) on whole population bins against SILVA SSU (small subunit) ribosomal RNA sequences (v119 release), and 23S rDNA matches were determined by using a blast search (word size = 18) on whole population bins against SILVA LSU (large subunit) ribosomal RNA sequences (v119 release). Only matches that were greater than 100bp in length are displayed.
